# JED: a Java Essential Dynamics Program for comparative analysis of protein trajectories

**DOI:** 10.1186/s12859-017-1676-y

**Published:** 2017-05-25

**Authors:** Charles C. David, Ettayapuram Ramaprasad Azhagiya Singam, Donald J. Jacobs

**Affiliations:** 1 Department of Bioinformatics and Genomics, University of North Carolina, Charlotte, USA; 2 Department of Physics and Optical Science, University of North Carolina, Charlotte, USA; 3 Center for Biomedical Engineering and Science, University of North Carolina, Charlotte, USA; 4 Current Address: The New Zealand Institute for Plant & Food Research, Limited, Lincoln, New Zealand

**Keywords:** Essential dynamics, Principal component analysis, Distance pairs, Partial correlations, Vector space comparison, Principal angles

## Abstract

**Background:**

Essential Dynamics (ED) is a common application of principal component analysis (PCA) to extract biologically relevant motions from atomic trajectories of proteins. Covariance and correlation based PCA are two common approaches to determine PCA modes (eigenvectors) and their eigenvalues. Protein dynamics can be characterized in terms of Cartesian coordinates or internal distance pairs. In understanding protein dynamics, a comparison of trajectories taken from a set of proteins for similarity assessment provides insight into conserved mechanisms. Comprehensive software is needed to facilitate comparative-analysis with user-friendly features that are rooted in best practices from multivariate statistics.

**Results:**

We developed a Java based Essential Dynamics toolkit called JED to compare the ED from multiple protein trajectories. Trajectories from different simulations and different proteins can be pooled for comparative studies. JED implements Cartesian-based coordinates (cPCA) and internal distance pair coordinates (dpPCA) as options to construct covariance (Q) or correlation (R) matrices. Statistical methods are implemented for treating outliers, benchmarking sampling adequacy, characterizing the precision of Q and R, and reporting partial correlations. JED output results as text files that include transformed coordinates for aligned structures, several metrics that quantify protein mobility, PCA modes with their eigenvalues, and displacement vector (DV) projections onto the top principal modes. Pymol scripts together with PDB files allow movies of individual Q- and R-cPCA modes to be visualized, and the essential dynamics occurring within user-selected time scales. Subspaces defined by the top eigenvectors are compared using several statistical metrics to quantify similarity/overlap of high dimensional vector spaces. Free energy landscapes can be generated for both cPCA and dpPCA.

**Conclusions:**

JED offers a convenient toolkit that encourages best practices in applying multivariate statistics methods to perform comparative studies of essential dynamics over multiple proteins. For each protein, Cartesian coordinates or internal distance pairs can be employed over the entire structure or user-selected parts to quantify similarity/differences in mobility and correlations in dynamics to develop insight into protein structure/function relationships.

**Electronic supplementary material:**

The online version of this article (doi:10.1186/s12859-017-1676-y) contains supplementary material, which is available to authorized users.

## Background

Many simulation techniques are available to generate trajectories for sampling protein motion [1–3]. Molecular conformation is represented by a vector space of dimension equal to the number of degrees of freedom (DOF). Investigating a trajectory in terms of a set of selected DOF can help understand protein function. The DOF are usually Cartesian coordinates that define atomic displacements. Internal DOF can also be employed, such as *distances* between *pairs* of carbon alpha atoms [4, 5]. Distance pairs simplify the characterization of protein motion, and can often be measured experimentally [6]. The process of extracting information from an ensemble of conformations over a trajectory is a task well suited for statistical analysis. Specifically, principal component analysis (PCA) is a method from multivariate statistics that can reduce the dimensionality of the DOF through a decomposition process to quantify essential dynamics (ED) [7] in terms of collective motions [5, 8, 9].

PCA is a linear transformation of data that extracts the most important aspects from a covariance (Q) matrix or a correlation (R) matrix. The R-matrix is obtained by normalizing the Q-matrix. When the property of interest is variance, statistically significant results from Q are skewed toward large atomic displacements. When the objective is to identify correlated motion without necessarily large amplitudes, the R-matrix should be used. For example, if the swinging motion of two helixes are highly correlated with the amplitude of one helix 1/10 that of the other, covariance will likely miss this correlation. In constructing a Q- or R-matrix it is best to have sufficient sampling, and to mitigate the problematic skewing effect of outliers [10, 11].

Eigenvalue decomposition calculates eigenvectors, each with an eigenvalue, that define a complete set of orthogonal collective modes. Larger eigenvalues for Q or R respectively describe motions with larger amplitude or correlation. Eigenvalues from the Q-matrix are plotted against a mode index sorted from highest to lowest variance. A “scree plot” typically appears indicating a large fraction of the protein motion is captured with a small number of modes. These modes define an “essential subspace” thought to govern biological function. For the R-matrix, modes with eigenvalues greater than 1 define statistically significant correlated motions. The projection of a conformation onto an eigenvector is called a principal component (PC). A trajectory can be subsequently described in terms of displacement vectors (DV) along a small number of PC-modes to facilitate comparative studies where differentiation in dynamics may have functional consequences.

To quantify large-scale motions of proteins PCA has been commonly employed [12–14]. The cosine content of the first principal component is a good indicator of the convergence of a molecular dynamics simulation trajectory [15]. Cartesian PCA (cPCA) and internal coordinate PCA methods are frequently used in characterizing the folding and unfolding of proteins [16, 17] and understanding the opening and closing mechanisms within proteins, including ion channel proteins [18–21]. More generally, PCA is routinely employed to elucidate the variance in the distribution of sampled conformations in a molecular dynamics trajectory [22]. Conformational dynamics of a protein upon ligand binding has also been investigated with a PCA approach [23]. With continual increase in computational power and commonly employed coarse-grained models [24–26] it is now feasible for a typical lab to perform comparative studies that involves the analysis of many different molecular dynamics trajectories. Such studies of interest include structure/function scenarios that interrogate the effects of mutation on protein dynamics, allosteric response upon substrate binding, comparative dynamics across protein families under identical solvent/thermodynamic conditions, change in conformational dynamics under differing solvent/thermodynamic conditions or different bound substrates. For example, in our previous work in studying myosin V [5, 6, 27], where we compared various apo versus holo and wild-type versus mutant systems motivated building a general tool to handle comparisons of dynamical metrics across different protein systems. When applied on a collection of systems, PCA extracts similarities and differences quantitatively.

When scaling up to analyze a collection of molecular dynamics trajectories, a toolkit to conveniently perform a comprehensive set of operations is needed. Hence, we designed JED (Java Essential Dynamics) as an easy to use package for PCA applied to Cartesian coordinates (cPCA) and distance pairs (dpPCA). While JED makes the analysis of a single protein trajectory straight forward with lot of built in features, it also allows the same features to be leveraged on a collection of trajectories to perform comparative analysis. The features JED offer are: **(1)** outlier removal; **(2)** creates Pymol scripts to visualize individual PC-modes and essential motion over user-selected time scales as movies; **(3)** creates free energy surfaces for two user-selected PC-modes based on Gaussian kernel density estimation; **(4)** calculates the precision matrix from Q and **(5)** the partial correlation matrix (P) along with its eigenvectors and eigenvalues; **(6)** compares the essential dynamics across multiple proteins and quantifies overlap between vector subspaces, and **(7)** multivariate statistical analysis methods are holistically utilized.

## Methodology

A dynamic trajectory provides snapshots (frames) depicting the various conformations of a protein. For Cartesian PCA (cPCA) the set *A*_*raw*_ = {*X*(*t*)} where *t* is a discrete variable refers to a particular frame. The vector *X* describes the position vectors of a user-selected set of alpha carbon atoms within the protein. For *m* residues, *X* is a column vector of dimension 3*m* since there are (*x*, *y*, *z*) coordinates for each alpha carbon atom. For *n* observations, *A* is a matrix of dimension 3*m* × *n*. To study internal motions, the center of mass of each frame is translated to the origin, and each frame is rotated to optimally align its orientation to the reference structure, *X*_*ref*_, which also has its center of mass at the origin. We use a quaternion rotation method to obtain optimal alignment, which yields the minimum least-squares error for displacements between corresponding atoms [5].

**Fig. 1:**
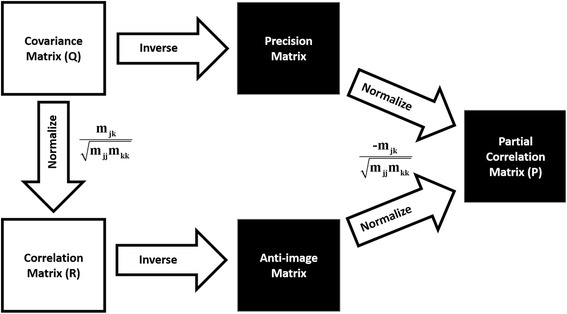
Full circle of R, Q and P matrix calculations

Since the process of translation and rotation changes the coordinates of each frame, the transformed *A* matrix is denoted as *A*_*Aligned*_ = {*X*_*Aligned*_(*t*)}. The reference structure *X*_*ref*_ is user specified in JED (selecting the initial structure is common practice). The data in matrix *A*_*Aligned*_ is mean centered along its rows to arrive at *A* '. The covariance matrix *Q* associated with *3 m* variables is defined as *Q* = *A* ' *(A')* ^*T*^ , which is real and symmetric, and has dimension 3*m* × 3*m*. If *n* ≥ 3*m*, the eigenvector decomposition of *Q* will have 3*m* − 6 non-zero eigenvalues, where 6 zero eigenvalues correspond to the modes of trivial degrees of freedom (3 for translation and 3 for rotation). The same is true for the correlation matrix *R*. In building a *Q* - or *R*-matrix, JED removes outliers based on a user-defined threshold. In practice, no zero eigenvalues occur due to alignment variations, which means the condition number of the Q and R matrices is finite, and both matrices have an inverse. The partial correlation matrix P is calculated by normalizing the inverse of Q. Figure 1 shows how R, Q and P are calculated. The procedure for distance pair PCA (dpPCA) is mathematically identical. However, dpPCA does not require the alignment step described above because internal distances are invariant under translation and rotations.

## Implementation

The Java code for JED can be downloaded from (https://github.com/charlesdavid/JED). Additional resources are provided regarding PCA, essential dynamics, example datasets together with example JED input files. JED is written in Java and implements the JAMA Matrix package and calls the KDE (https://github.com/decamp/kde) to perform the following tasks:

1. The file JED_Driver.txt is input to JED to define all information needed to run a job. The file PDB_Read.log lists all PDB files processed in the order read. The “JED_LOG.txt” file summarizes how the run progressed. Details about output file formats and how to setup JED_Driver.txt is documented in a User Manual (given in Additional file 1).
2. Reads in sets of PDB files (or coordinate matrix files constructed by JED).
  - The PDB files may be single chain or multi chain.
3. The program performs analysis at the coarse-grain level of all alpha carbons.
4. The user can select a subset of residues for the analysis that need not be contiguous.
  - In multi chain PDBs, the residues may come from the various chains.
5. As an initial pre-PCA output, the following characteristics are determined:
  - Matrix of atomic coordinates before and after the optimal alignment is performed.
  - Conformation RMSD and residue RMSD otherwise known as RMSF.
  - The B-factors in a PDB file are replaced with residue RMSD.
6. The user can run cPCA, dpPCA or both.
7. The user can choose the number of most relevant modes to retain.
8. The user can specify a z-score cutoff (a decimal ≥ 0) such that when the value of a PCA variable (either a Cartesian or internal distance coordinate) has a |deviation| from its mean that exceeds the z-score cutoff, it is identified as an outlier. When the value of a variable is identified as an outlier, it is replaced by its mean value. This process is done per variable, per frame, treating each variable independently. This method is recommended because it reduces condition numbers on Q, R and P, with little loss in statistics to avoid misinterpreting the PCA results. However, an option is provided for the commonly used alternative that throws out conformations that have a RMSD value deemed as an outlier.
9. All quantitative metrics are outputted as text-files for further analysis and graphing. For both cPCA and dpPCA the following characteristics/metrics are determined:
  - The displacement vectors (DV).
  - The covariance (Q), correlation (R) and partial correlation (P) matrices.
  - All eigenvalues for Q, R and P.
  - Three sets of the most relevant PC modes coming from Q, R and P.
  - Weighted and unweighted mean squared fluctuation (MSF) and root mean squared fluctuation (RMSF) for all three sets of the most relevant PC modes are provided.
  - For cPCA, a set of PDB files and associated Pymol scripts allow static pictures and movies of the 3D structure to be viewed for each set of the most relevant PC modes.
10. DV projections onto each of the most relevant eigenvectors (weighted and unweighted).
11. Multiple jobs can be run using the same set of parameters using a batch driver.
12. Essential motions from Q, R and P results can be generated for any user-selected window of PC-modes, corresponding to observing protein motions on different time scales.
13. After each individual trajectory is processed, additional programs can be run to perform a comparative analysis. These programs are:
  - Create_Augmented_Matrix.java: Pools together multiple trajectories into a single dataset to facilitate another JED analysis on the collection of data.
  - Subspace.java: Runs comparisons between individual trajectories and/or a pooled trajectory. The outputs are cumulative overlaps (CO), root mean square inner product (RMSIP), and principal angles (PA).
  - Get_FES.java: Creates a free energy surface for any two user-selected PC-modes.
  - VIZ_Driver.java: Allows control for animating motions for individual PCA modes and combined superposition of essential PC modes related to timescale windowing.

The R and P matrices are computed from Q. The Q, R and P matrices are stored in memory (order O^2^) and then diagonalized (order O^3^) for a complete eigenvalue decomposition using the JAMA matrix package. For 2000 frames of a 250 residue protein the performance time on a modern laptop is less than 3 min. For comparative studies, similarity of conformational ensembles is quantified in terms of the vector subspaces that characterize ED. JED calculates cumulative overlap (CO), root mean square inner product (RMSIP), and principal angles (PA) [28–32]. Overlapping subspaces from different proteins imply they share similar dynamics, whereas different protein motion is indicative of subspaces with low overlap.

## Results and Discussion

First, we show cPCA results describing ED of a protein. Second, we show dpPCA results, demonstrating how internal motions among different loops are easily quantified. Third, we show how pooling trajectories (using dpPCA) facilitates a comparative analysis of protein dynamics. As an illustrative example, a native single chain variable fragment (scFv) of 238 residues is considered, along with a mutant differing by a single site mutation (G56V). We work with a 100 ns molecular dynamics simulation trajectory for the native and mutant structures, each having 2000 frames taken from our previous study [33].

### Native and Mutant Essential Dynamics from cPCA

**Fig. 2:**
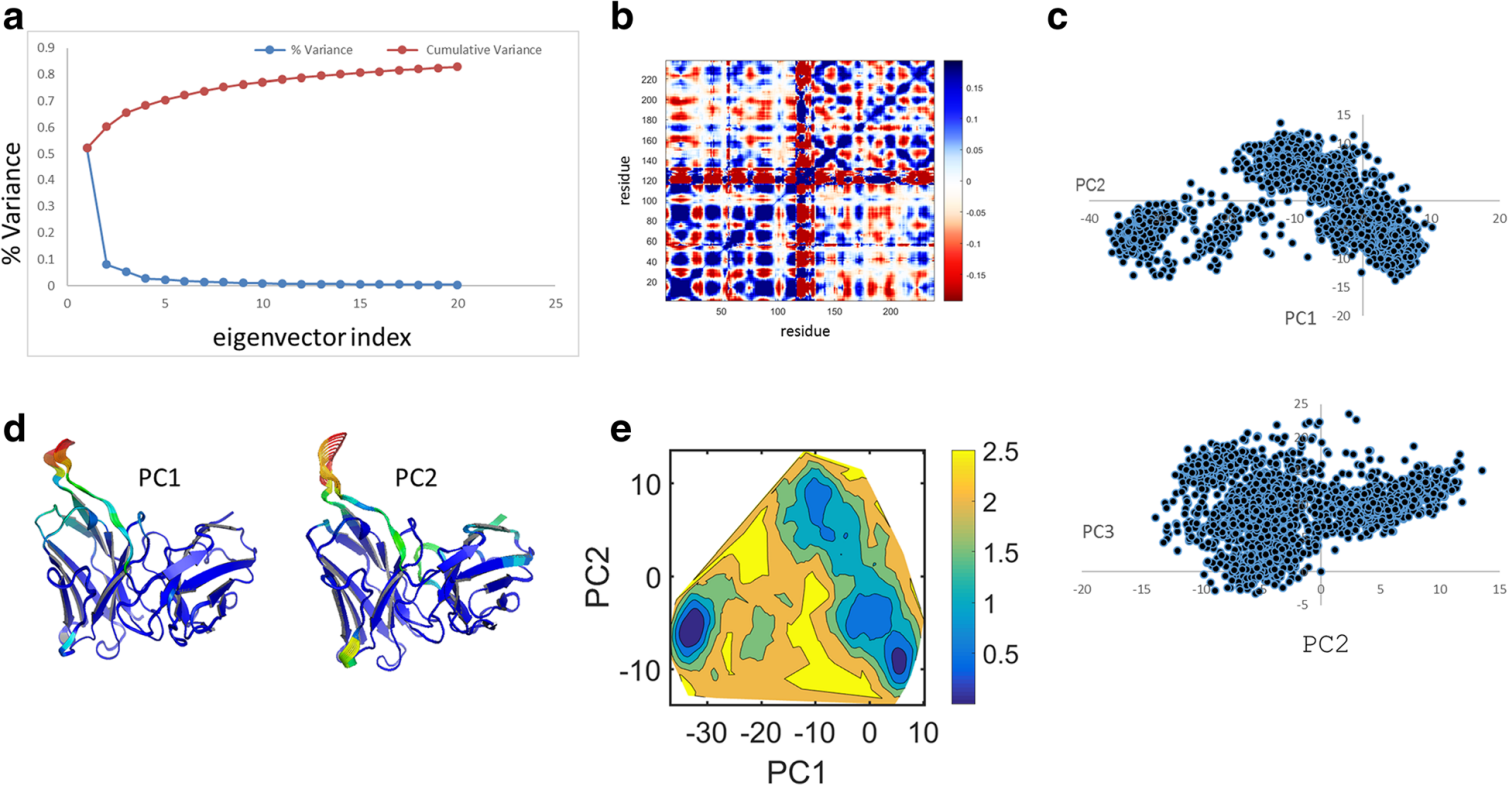
Some cPCA results for the native protein. **a** The variance and cumulative variance of the first twenty principal components. **b** The reduced Q-matrix. **c** Projections of the trajectory onto the planes formed by (PC1 and PC2) and (PC2 and PC3). **d** The displacements along PC1 and PC2 are visualized and colored according to their RMSF for each residue using Pymol^™^. **e** The free energy surface associated with the first two principal components

**Fig. 3:**
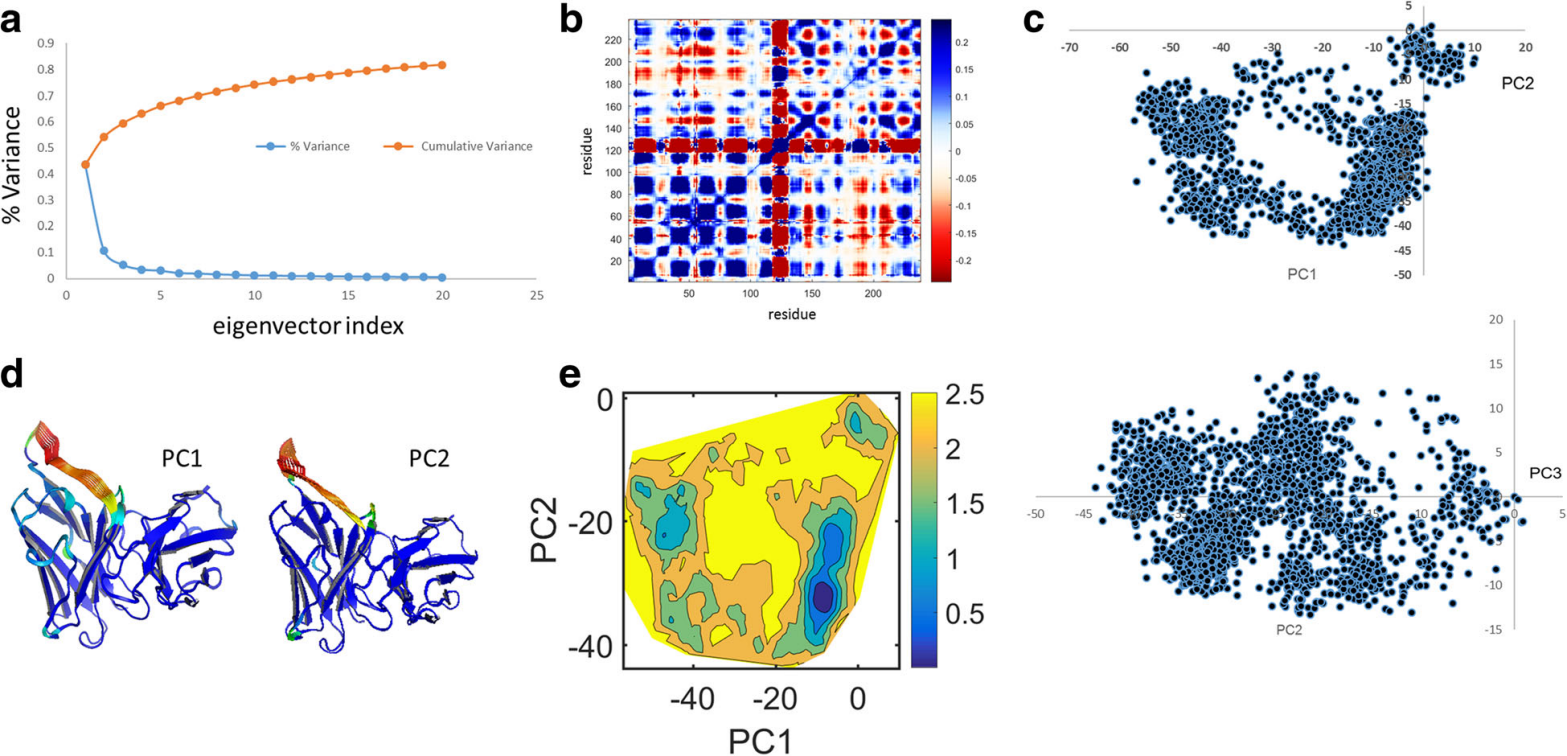
For the mutant protein the same type of cPCA results are shown as in Fig. 2. **a**) Thevariance and cumulative variance of the first twenty principal components. **b**) The reduced Q-matrix. **c**) Projections of the trajectory onto the planes formed by (PC1 and PC2) and (PC2 and PC3). **d**) The displacements along PC1 and PC2 are visualized and colored according to their RMSF for each residue using Pymol™. **e**) The free energy surface associated with the first two principal components

To characterize the ED of the native and mutant (G56V) proteins we performed cPCA on their trajectories. We show multiple output types in Figs. 2 and 3 for the native and mutant proteins respectively. For convenience in understanding the role of correlations, JED also outputs the reduced Q-matrix defined as ${\tilde{Q}}_{jk}={Q}_{xj, xk}+{Q}_{yj, yk}+{Q}_{zj, zk}$. Here, the j and k indices label residues, and the original 3*m* × 3*m* covariance matrix is transformed into a rotationally invariant *m* × m matrix, which is common practice. Figures 2a and 3a show that the first 20 eigenvectors are most informative and shows maximum variation of 80% of the total variance. The reduced Q-matrix (Figs. 2b and 3b) shows which pairs of residues move together as positive correlation (blue) and away from one another as negative correlation (red). It can be seen that the native protein (Fig. 2b) has more anticorrelated motions between the residues when compared to that of the mutant system (Fig. 3b). All other 3*m* × 3*m* matrix types have a reduced version, with both format types outputted by JED. The projection of PC1 vs PC2 and PC2 vs PC3 for native and mutant are shown in Figs. 2c and 3c respectively. The trace values for the native and mutant structures are 432 Å^2^ and 644 Å^2^ respectively. The larger value for the mutant suggests that there is an overall increase in flexibility of the mutant. For a particular PC mode, 3D ribbons depicting protein structure are colored by the RMSF to show mobility where high to low values are colored by red to blue as shown in Figs. 2d and 3d for native and mutant system respectively. The free energy surface (FES) obtained from the first two principal components for native and mutant proteins are shown in Figs. 2e and 3e respectively. In these examples, the free energy landscape for the native protein has two well-defined basins, while for the mutant it has only one basin and the conformations were scattered due to the increased in flexibility.

JED provides similar output for the R- and P-matrices. In Additional file 2: Figures S1 and S2 show results for the R-matrix. Differences seen within the first two PC modes indicate in part how the G56V mutant perturbs protein motion. Comparing the results from the covariance and correlation matrices show that the former highlights the most dramatic motions, while correlations among low amplitude motions is largely missed. Additional file 2: Figures S1 and S2 on the other hand show that there is a much greater richness in correlations in conformational changes when the amplitude of motion is not allowed to be the dominant characteristic in the analysis. We recommend that a user should analyze results from the Q- and R-matrices because they capture different correlated motions with different amplitude scales. In this example, the R-matrix results uncover subtle collective motions without an associated large amplitude motion, which may have functional consequences and are more sensitive to mutation. Both types of output provide insight about potential mechanisms that govern protein dynamics. Movies for PC-modes obtained from the Q and R matrices are given in Additional file 3. To quantify the similarity in the ED retained in the top PC modes from the Q-, R- or P-matrices, JED calculates overlap in these vector spaces. This feature allows one to access how much shared information there is between using different metrics, as well as between different molecular dynamics trajectories. Results for RMSIP and PAs over 20 most essential dimensions are shown in SI in Additional file 2: Table S1. Because up to 30 degrees in PA constitutes high similarity, Additional file 2: Table S1 shows that 6 PC modes are needed to capture ED accurately. With 6 PC modes the cumulative variance covers ~74% or ~70% of the dynamics for the native and mutant protein respectively. Note that 70% cumulative variance is a commonly used criterion to decide the number of PC modes to keep. A subspace comparison between the native and mutant proteins in terms of PA and RMSIP is made in SI where Additional file 2: Figure S3 and Table S2 reveals similar dynamics is described with 11 PC modes. Therefore, the native and mutant proteins exhibit the same ED. In SI, Additional file 2: Figures S4 and S5 show results for the P-matrix. In addition to the R and P matrices, JED outputs their inverses, which are respectively called precision and anti-image matrices (see Fig. 1).

### Visualization of Essential Protein Motion

The protein motion that is expected to be important for biological function constitutes a linear superposition of PC-modes from the essential subspace. Because protein dynamics spans a large range in time scales, JED allows essential protein motion to be visualized within a window of time scales by combining PC-modes over a user-selected set of PC-modes given by:

$$ \overrightarrow{X}\left(\tau \right)={\displaystyle \sum_{k={k}_o}^{k_o+ w}}{A}_k \sin \left({\omega}_k\tau \right){\overrightarrow{V}}_k $$

where *τ* is the time of the movie, ${\overrightarrow{V}}_k$ is the k-th PC-mode with *λ*_*k*_ its eigenvalue, ${A}_k= C\sqrt{\lambda_k}$ and ${\omega}_k= B\sqrt{\frac{1}{\lambda_k}}$ for the Q and R matrices, while ${A}_k= C\sqrt{\frac{1}{\lambda_k}}$ and ${\omega}_k= B\sqrt{\lambda_k}$ for the P-matrix. Here, B and C are constants adjusted to set appropriate time and space scales respectively. The index *k*_*o*_ defines the starting PC-mode (often equal to 1) and *w* is the window size. Watching movies at different time scales gives a sense of the effects of small and large amplitude motions (see Additional file 3 for movies of essential motions of the ScFv protein over different windows). In this case, the movies show the mutation rigidifies nearby residues in corroboration with our previous results [34]. To our knowledge, visualizing combination of modes within user-specified time scale windows offers a unique functionality/tool for researchers.

### Reduction of Dimensionality by dpPCA

JED utilizes internal coordinates based on residue-pair distances (**dpPCA**). A user selects *n* residue-pairs, where a carbon-alpha atom defines the motion of a residue. The dimensionality of the Q-matrix is therefore *n*. When *n* is much less than the number of residues, the reduction in DOF also reduces noise to signal. Importantly, dpPCA allows intuition to be used when deciding which distance pairs to consider. Distance-pairs can be placed between residues having aligned positions based on sequence or structure. This facilitates dynamics of homologous proteins to be directly compared. In the example used here, a single site mutation retains the protein size with perfect alignment. We select distance pairs from the loop regions (H1, H2, H3, Linker, L1, L2, L3) to residue 56 for the native and mutant proteins, which gives *n* = 74 (see Additional file 2: Figure S6 in SI).

**Fig. 4:**
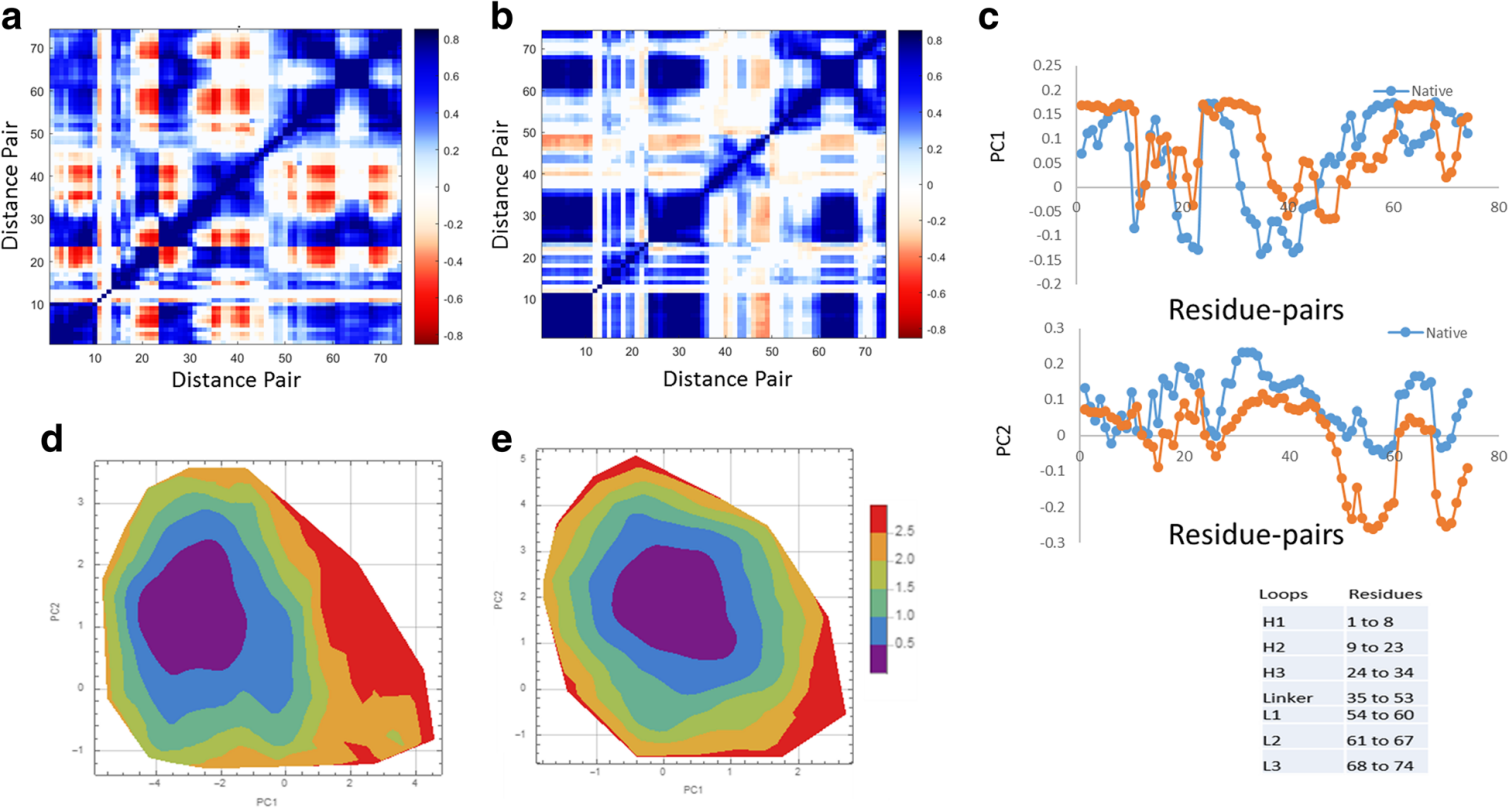
**a** and **b** Correlation (R) Matrix as obtained from the dpPCA of native and mutant respectively. **c** Comparing the 1^st^ and 2^nd^ PC modes for the native and mutant proteins. **d** and **e** Free energy surface obtained from the top two PC modes for the native and mutant respectively

**Fig. 5:**
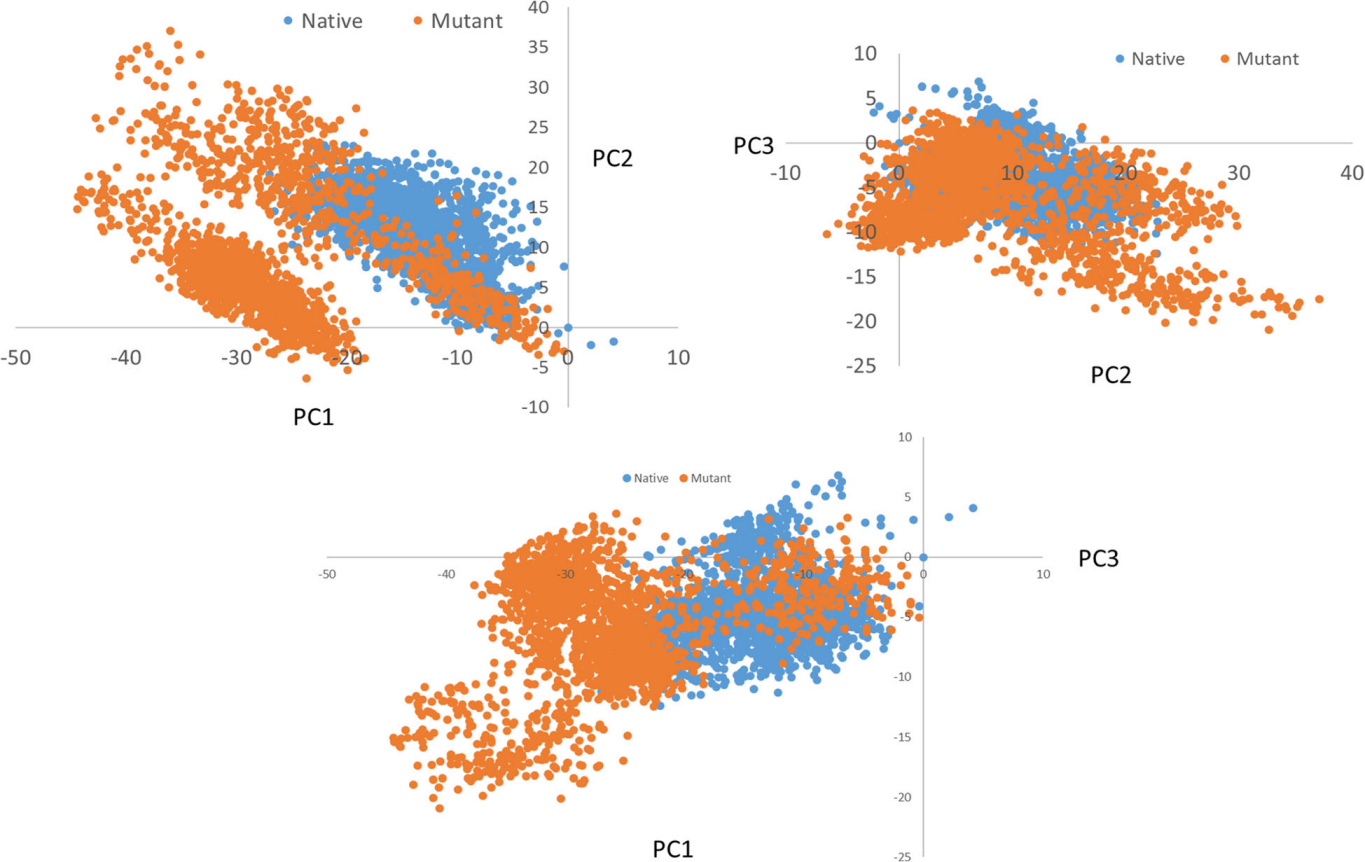
PCA scatter plot along the pair of different combinations of first three pair combinations of principal components (PC1, PC2 and PC3)

The dpPCA R-matrix is shown in Figs. 4a and 5b where differences in correlations within the native and mutant proteins appear. Figure 4c shows the PC-modes of distance pairs, which describe how distances between residues stretch or contract. From the figures, we can clearly see some difference in dynamics of native and mutant. From Additional file 2: Tables S3, S4 and Figure S7 in SI, at least 6 PC modes are needed to characterize the dynamics of the loops relative to residue 56. Because similarities in motion between the native and mutant proteins extend up to 15 PC modes, the ED of this set of distance pairs is the same between the native and mutant proteins. The free energy surface defined by the PC1 and PC2 modes (see Fig. 4d and e) are similar for the native and mutant proteins. In general, projecting trajectories onto a plane (a two-dimensional subspace) within a high dimensional vector space leaves open a likely possibility that the projections are not common to the same plane. In Additional file 2: Table S5 in SI, it is seen that the planes describing the top two PC modes (from the R-matrix) for the native and mutant proteins are very similar, somewhat justifying the direct comparison of free energy surfaces using PC1 and PC2 from two different calculations. However, Additional file 2: Table S5 also shows that the first two PC modes for the Q-matrix (from the native and mutant proteins) are not similar, which is in part a reason why free energy surfaces appear different in Additional file 2: Figure S8.

### Comparative Analysis by Pooling Trajectories

For comparative studies, it is necessary to use the same set of coordinates. JED facilitates this by allowing a user to pool trajectories together. In order to compare the difference between the native and mutant, we combine native and mutant trajectories and calculate dpPCA on the selected subset defined above where no alignment required for dpPCA. Pooling is also possible with cPCA with an alignment step. Figure 5 shows a scatter plot of different combination of PCs (PC1- PC2 (Fig. 5a), PC1-PC3 (Fig. 5b) and PC2-PC3 (Fig. 5c)) depicting a significant difference between the two systems. In particular, it is evident from the figure that the mutant occupies a larger phase space and exhibits a higher fluctuation compared to the native, which implies that the mutant has a higher degree of mobility when compared to native. It is also possible to obtain FES for any two PC from JED using JED_get_FES.java. FES for different combinations of PCs is given in SI in Additional file 2: Figure S9.

## Conclusions

We have developed an essential dynamics analysis package written in Java that performs a complimentary set of tasks following best practices for multivariate statistics. The JED toolkit offers much more functionality compared to currently available tools. Particularly unique aspects of JED are the Z-score based elimination of outliers, distance pair PCA (dpPCA), convenient comparative analysis of subspaces using principal angles, visualization of essential motions, and the inclusion of the full circle of statistical metrics that include precision matrices and the partial correlation matrix. The program can be run from a compiled source or from executable jar files. Additional resources that can be downloaded with the program include example test cases with all JED results and a detailed user manual, which is also included in SI as a PDF.

## Additional files

Additional file 1: User Manual and Tutorial for the JED package. (PDF 408 kb)

Additional file 2: Supporting Information. **Figure S1**. Example results from cPCA using the R matrix for native. **Figure S2**: Example results from cPCA using the R matrix for mutant. **Figure S3.** Subspace comparison between native and mutant cPCA results **Figure S4**: Example results from cPCA using the P matrix for native. **Figure S5**: Example results from cPCA using the P matrix for mutant. **Figure S6**: Selection of residue-pair distances. **Figure S7.** Subspace comparison between native and mutant dPCA results. **Figure S8:** Example results from dPCA using the Q matrix for native and mutant. **Figure S9**: Free energy surfaces based on all pairwise combinations of the top three PC-modes based on pooling the native and mutant trajectories. **Table S1:** Subspace comparison between all possible pairs of Q-, R- and P-matrices using cPCA for native and mutant. **Table S2:** A twenty dimensional subspace comparison between native and native for each of the Q-, R- and P-matrices using cPCA. **Table S3:** A twenty dimensional subspace comparison between all possible pairs of Q-, R- and P-matrices using dPCA for native and mutant. **Table S4:** A twenty dimensional subspace comparison between native and native for each of the Q-, R- and P-matrices using dPCA. **Table S5:** Same as Table S4 expect a 2 dimensional subspace is being compared. (PDF 4690 kb)

Additional file 3: Movies showing mode 1 and mode 2 of all the modes obtained from the JED program. (PPT 58883 kb)

## Abbreviations

cPCA: Cartesian Principal component analysis
dpPCA: Distance pair Principal component analysis
DV: Displacement vectors
JED: Java Essential Dynamics
P: Partial Correlation matrix
PC1: Principal component 1
PC2: Principal component 2
PC3: Principal component 3
Q: Covariance Matrix
R: Correlation matrix
