# Supplementary material for: JED: a Java Essential Dynamics Program for comparative analysis of protein trajectories
Source: BMC Bioinformatics. 2017 May 25;18:271. doi: 10.1186/s12859-017-1676-y (PMC5445469; doi:10.1186/s12859-017-1676-y)
Supplement: Supplementary file 1 — User Manual and Tutorial for the JED package. (PDF 408 kb) [file 12859_2017_1676_MOESM1_ESM.pdf]

JED: A Java Essential Dynamics Program for Comparative Analysis of Protein Trajectories

Charles C. David[1†]*, E. R. Azhagiya Singam[2] and Donald J. Jacobs[2]*

[1]Department of Bioinformatics and Genomics, [2]Department of Physics and Optical Science, University of North Carolina at Charlotte, USA

†Current Address: The New Zealand Institute for Plant & Food Research Limited, Lincoln, NZ

*Correspondence: charles.david@plantandfood.co.nz or djacobs1@uncc.edu

Java Essential Dynamics (JED)

User Manual and Tutorial

Acknowledgement

Dr. Charles David wrote the majority of the JED program during his Ph.D. studies in Bioinformatics and Computational Biology at UNC Charlotte under the direction of Dr. Donald Jacobs. Partial support for this work came from NIH grants (GM073082 and HL093531), from the Center of Biomedical Engineering and Science, and the Department of Physics and Optical Science.

GNU General Public License Agreement

If you choose to use JED software, you agree to cite the references listed below on all publications that present results based on the JED analysis, and you agree to abide by the GNU General Public License Agreement (version 3). The GNU General Public License Agreement can be found at http://www.gnu.org/licenses/gpl.html

Citation

Charles C. David, E. R. Azhagiya Singam and Donald J. Jacobs. *JED: A Java Essential Dynamics Program for Comparative Analysis of Protein Trajectories* BMC (publication information, link).

Table of Contents:

- **I. Introduction** 2
- **II. Using JED** 4
- **III. Overview of Using JED** 6
- **IV. Using JED Driver** 8
  - **A. The Preliminary Run** 8
  - **B. Debugging Crashes Part I** 10
  - **C. Performing Only cPCA** 11
  - **D. Performing Only dpPCA** 16
  - **E. Debugging Crashes Part II** 20
  - **F. Performing Cartesian Mode Visualization** 21
  - **G. Performing Multiple PCAs** 22
- **V. Using JED Batch driver** 23
  - **A. JED Batch Driver Input File Format: The Preliminary Run** 23
  - **B. JED Batch Driver Input File Format: Only cPCA** 24
  - **C. JED Batch Driver Input File Format: Only dpPCA** 25
  - **D. JED Batch Driver Input File Format: Combined cPCA, dpPCA, VIZ** 26
  - **E. Debugging Batch Crashes** 26
- **VI. Additional Types of Analysis** 27
  - **A. Pooling Data** 27
  - **B. Subspace Analysis** 28
  - **C. Free Energy Surface** 29
  - **D. Essential Subspace Visualization** 31
- **Appendix** 33
  - **I. Input File Formats** 33
  - **II. Output File Formats** 36

I. INTRODUCTION

Java Essential Dynamics (JED) is a java library (a package of programs) for analyzing protein trajectories. The trajectories may be derived from any molecular dynamic simulation method that outputs a trajectory as a set of PDB files. The program can handle single chain PDB files with no chain identifier as well as multi chain PDB files that use chain IDs. The user may specify the set of residues to be considered for the analysis, and this set need not be contiguous. A variety of utility tools related to **Principal Component Analysis** (PCA) provide users with additional features not found in MD-simulation packages. This stand-alone statistical software package is well suited for quantitatively comparing differences in protein dynamics. In particular, JED provides convenient tools to help with comparative analysis of protein dynamics from multiple trajectories. JED is capable of running on any platform with a suitable Java Runtime Environment (JRE).

Expected Input to JED:

Ideally, each PDB structure should follow standard PDB-format, although deviations from standard often work fine. The first residue label must start at 1 or higher. No 0 or negative numbers are allowed for residue labels. Preprocessing of PDB files should be done with external software that generates the conformational ensembles before using JED. It is convenient and recommended to label PDB files using leading zeros in the name of the files to simplify tracking time progression. For example, if a simulation generates 100,000 frames in the trajectory, it is best to name the PDB files like <file_name_000000>, <file_name_000001>, … <file_name_100001>. In this way, all frames are specified relative to the starting structure in sequential order.

JED Preprocessing Output:

As a preprocessing step, JED reads in all PDB files in a specified directory and aligns all the structures in the trajectory to a specified reference structure using a quaternion alignment algorithm. A matrix of the **read PDB coordinates,** obtained from all the residues in the input PDB files, is created so that it can be used for all subsequent JED runs. A list of all the residues (**residue list**) found in the PDB files (along with the chain IDs when appropriate) is generated. The original and transformed **conformation RMSD** are determined for each member structure in the trajectory relative to the specified reference structure. The **residue RMSD** (also commonly referred to as **RMSF**) is determined from the entire trajectory. An **edited PDB file** is also generated where the B-factors are replaced with the **residue RMSD** values for visualization purposes. The Z-scores for the variables are also calculated. This output automatically happens and is non-optional.

Carbon Alpha Atoms:

The current implementation of JED only considers Cα atoms. As such, we speak about residues because the information is tied to Cα atoms that represent dynamics of residues at a coarse grained level of description. For example, the distance between two residues is modeled in JED as the distance between the two Cα atoms associated with the two residues. By working only with Cα atoms allows the Cα atom labels to be synonymous with residue labels. For a single chain protein, this is a simple 1 to 1 mapping. For multiple chain proteins, JED also tracks the chain ID.

Different Types of PCA:

The core element of essential dynamics is to perform PCA. JED implements two variations of PCA. The first and most common method is based on Cartesian coordinates (**cPCA**). The cPCA using ***n* residues** will yield eigenvectors having ***3n*** components, each corresponding to one Cartesian coordinate. The second method is based on internal coordinates using residue-pair distances (**dpPCA**). The dpPCA, using ***n* residue-pairs,** will yield eigenvectors having ***n*** components, each corresponding to one of inter-residue distance pairs. As a special case, an all-to-all comparison can be performed. However, an all-to-all comparison is computationally intense unless a small subset of residues is being considered.

PCA Models:

PCA methods are performed using a **covariance matrix** (**Q**), a **correlation matrix** (**R**) and a **partial correlation matrix (P)**. The correlation matrix is a normalized version of the covariance matrix. The results obtained from **Q** and **R** generally differ somewhat due to the inherent statistical biases in each approach. The **P** matrix is obtained from the inverse of the

covariance matrix, with is then subsequently normalized. The current implementation automatically considers all three types of statistical metrics, and allows these metrics to be compared.

Conditioning of the sample Q Matrix:

JED has functionality to remove outliers prior to PCA using two approaches. First, the user can specify the percent (a decimal [0, 1]) of outlier structures to be removed from the sample based on conformation RMSD. The most deviant structures are tagged as outliers and subsequently removed from the sample. Although not the recommend approach because it discards massive amounts of data, it is included in JED because it is a commonly used method. Second, the user can specify a z-score cutoff (a float > 0) such that when the value of a PCA variable has a |deviation| from the variable mean that exceeds the z-score cutoff, it is identified as an outlier. For each PCA-entry identified as an outlier, it is replaced with its mean. This process is done per variable over all frames, and each PCA-entry is treated independently. This is the recommended method because a frame is never thrown away. Rather, only outlier entries (a small fraction of all variables) within a frame are modified in a way that preserves the mean. Both methods are intended to reduce the condition number of **Q** and to improve the estimator for the population covariance matrix. The first method of conditioning is often employed for protein dynamics (if at all). The second method of conditioning is commonly used in the field of statistics, and is the preferred method due to its superior effectiveness. Note that without conditioning, the results from PCA risk being highly skewed (having statistical bias) due to the presence of outliers. PCA results are always highly dependent on the quality of sampling. Therefore, it is strongly recommended to use the z-score cutoff conditioning method in all applications to avoid misinterpreting the PCA results. Since the **R** and **P** matrices derive from the **Q** matrix, this same conditioning process also improves the **R** and **P** matrices. To monitor the effect of outlier removal, different cutoffs should be considered and compared. Both outlier removal methods can be turned off independently simply by setting percent to 0 and/or z-score cutoff to 0.

Animated Visualization of cPCA modes:

JED computes the root mean square deviation (RMSD) and mean squared deviation (MSD) of cPCA modes without weighting, and by weighting the modes by their corresponding eigenvalue. The RMSD and MSD characteristics of cPCA modes can be animated directly on the protein 3D structures. The user can specify the number of Cartesian modes to animate, beginning with mode one. The animation of a mode is done by creating a set of 20 PDB files that capture the displacement of each residue's atoms for each requested mode using a sine function to produce atomic displacements in proportion to eigenvector components. A scale-factor parameter is used to control the amount of displacement in the modes. A PyMol™ script is generated to animate the frames. A movie for an individual mode looks like a vibrational mode having a sinusoidal periodic motion. A scale-factor parameter of 1 provides physically realistic levels of atomic displacements. However, the user may wish to increase the scale-factor to emphasize motions more clearly. Because the eigenvectors from dpPCA cannot be mapped to residue displacements a simple way, visualization is not provided.

Animated Visualization of Essential Dynamics:

JED provides a PyMol™ script to show movies for the superposition of PCA modes. It is assumed that the modes vibrate in phase through a time dependent sinusoidal function that governs the mode amplitude. Since the relative amplitude of higher frequency modes decrease rapidly, the user can select a window of modes (lowest to highest consecutively) to visualize the essential dynamics of the protein at different time scales, which is set by the lowest frequency mode in the window. A user specifies the first PCA mode # to define the leading edge of the window, along with the number of modes in the window. A good window size is usually 5 modes. The user can generate different movies for the essential dynamics at different time scales by sliding the window (say 5 modes in size). For example, on the slowest time scale, the user could select modes 1 through 5, while a selection from 16 to 20 would show a much faster time scale. JED by default provides an animation of the essential dynamics for the top N-modes, where N is *MIN(5,Ncalculated)*.

Dimension Reduction Level:

The primary purpose of applying PCA to capture the essential dynamics of a protein is to reduce the large dimension of variables to a much smaller number of variables that captures the greatest variance in protein motion. The **Q, R,** and **P**

matrices, once diagonalized, provide a set of eigenvalues and eigenvectors. The eigenvalues for proteins typically fall off fast for the first several modes, out of possibly thousands of modes. The number of dimensions needed to provide a fair assessment of the essential dynamics in a protein is system-dependent. The user can specify any number (say 20, which typically is more than needed) to obtain results for all possible selections, ranging from 1 up to the maximum value that is selected. As such, the user can see how the added dimensions help glean more information. Eventually, the user must decide the optimal number of dimensions to use for representing the essential dynamics based on one's objectives. For **Q**, **R** and **P** matrices, the eigenvectors with largest eigenvalues are deemed most important. The eigenvalues for **Q** and **R** are always positive, and they are always negative for **P**. It therefore is the case that the maximum eigenvalue from **P** has a magnitude that is always closest to zero.

Displacement Vectors:

A set of **displacement vectors** (**DV**s) based on the full conformational space is calculated using a specified reference structure. Those **DV**s are then projected onto a set of eigenvector directions to create delta vector projections (**DVP**s), which are <u>similar</u> to principle components (**PC**s). The **PC**s are delta vector projections, but according to the standard definition used in statistics, they are always relative to the mean conformation position as defined in the construction of the **Q, R,** or **P** matrix. In studying the essential dynamics of a protein, it is common to use a reference structure that has a particular physical or biochemical meaning, which is why we call these displacements **DVP**s, and not **PC**s. The DVPs are useful to visualize protein motions. For example, if the first two eigenvector directions are selected (those eigenvectors associated with the highest and second highest eigenvalues) the **DVP**s can be plotted for each frame to construct the trajectory in conformational space projected onto a two dimensional cross-section. Other eigenvector directions can be specified, allowing the user to investigate how the trajectory projects into the space defined by each eigenvector. The **DVP**s are given using normalized inner products, as well as weighted by the corresponding eigenvalue. The different methods highlight the structure of the data and provide scaling for visualization.

Post PCA Comparative Subspace Analysis:

JED performs a <u>s</u>ub<u>s</u>pace <u>a</u>nalysis (**SSA**) on the two equidimensional sets of eigenvectors generated from the **Q, R,** and **P** variants of PCA. The results provide a relative comparison for different subspace dimensions starting with 1 dimension up to the dimension chosen by the user (when selecting the number of Cartesian or Distance modes to process) in a sequential fashion. This allows one to quantitatively determine how different the PCA results are due to the choice of PCA model, while assessing the size of the essential subspace. Additional analysis can be done using driver programs from the subspace analysis class. To perform comparative tests, it is best practice to first generate equidimensional sets of eigenvectors from each trajectory of interest, as well as from a pooled trajectory to use as a reference set, while ensuring that the subsets of residues being analyzed are identical. Subspace analysis is done by comparing the sets of eigenvectors, directly or iteratively, and determining the root mean square inner products (**RMSIP**s), Principal Angles (**PA**s), cumulative overlap (**CO**s), cosine products (**CP**s), vectorial angular sum (**VAS**), and the maximum angle between subspaces of the given vector space. JED produces summary log files for all of these analyses.

II. <u>Using JED</u>

`Note: In this tutorial, code, file paths, and text file content are shown in dark blue 9 point Consolas`

JED Install Instructions:

Java is **platform independent** and JREs exist for all common architectures. JED requires **JRE version 1.7 or higher** installed. JED can be run from compiled source or from executable jar files. While JED can be installed in any directory that is part of your Java classpath, the source code must be compiled on the local machine to insure runtime integrity. When compiling from source, be sure to also compile the JAMA MATRIX (http://math.nist.gov/javanumerics/jama/) and KDE (https://github.com/decamp/kde) package as JED uses these library. Alternatively, no source code or compilation is needed to run the executable jar files. These can be placed in any directory that is on the Java classpath. Either the Java environment variable **CLASSPATH** should be correctly set to run Java programs at the command prompt, or add the **-cp** option to the **java** command, which allows you to specify the path that contains your Java classes.

Expected Memory Requirements:

For most applications, a **64bit OS is required** to address memory needs. On high performance computer clusters make sure the 64 bit JRE is installed. Memory use is demanding because JED loads the complete covariance matrix (among other data structures). Typically 8 to 32 GB of RAM is needed depending on the size of the protein. For very large proteins with thousands of residues and/or tens of thousands of frames, make available as much memory per node as possible. On most platforms, Java performance can be optimized by specifying parameters at runtime for heap space.

Two Kinds of JED Drivers:

There are two driver programs for JED: **JED_Driver** runs a single job using parameters specified in the input file, while **JED_Batch_Driver** runs a batch of jobs sequentially. The first is suited for running a single job at the command line or when using submit scripts on computer cluster resources. This can be implemented using job arrays so that your jobs run in parallel rather than sequentially. The second is suited for running multiple jobs on a single computer so that a user can submit a batch of jobs, perhaps overnight, and then come back later with all jobs finished without having to launch each one separately. It could be that a user will organize batch jobs in terms of similar conditions, such that it could make sense to run multiple batches in parallel on high performance clusters.

Note: The input file formats for the two driver programs are NOT equivalent.

Input File and Data for JED Driver:

JED requires an input file for job parameters. The format of this file will be described below. The run command takes only one argument, which is the name of the input file that includes the absolute path to the file. If no argument is specified, then JED assumes that the default input file name is used and the file is located in the same directory from which the Java Virtual Machine (JVM) was called. The default input file names are:

**JED_Driver.txt**	for JED_Driver.java (or .jar file)
**JED_Batch_Driver.txt**	for JED_Batch_Driver.java (or .jar file)

Each job should be assigned to its own directory, which must contain either the **PDB files** to read (for Pre-Processing runs) or the **Coordinates Matrix** to process (for all Analytical runs), along with the **reference PDB file** and **residue lists** for specifying the subsets of interest: Cartesian, and/or Distance Pairs.

JED Command Line format:

To run **JED_Driver** at the command prompt or within a PBS script, you can use one of the following commands:

```
java -d64 JED_Driver “/path/to/your/input/file.txt” (runs the compiled java program)
java -jar -d64 JED_Driver.jar “/path/to/your/input/file.txt” (runs the executable jar file)
```

To run **JED_Batch_Driver** at a command prompt or in a PBS script, you can use one of the following commands:

```
java -d64 JED_Batch_Driver “/path/to/your/input/file.txt”)
java -jar -d64 JED_Batch_Driver.jar “/path/to/your/input/file.txt”
```

Remember to include command line switches to optimize the Java runtime environment for your jobs.

Note: Different platforms may have slight variants to the options, such as –d 64 with a space, versus without a space.

Organization of Output Files:

Output files from JED are written to subdirectories within the working directory, structured to organize the multitude of files produced in a meaningful manner. The start of this directory tree (the root) is named "JED_RESULTS_**$description**", where **$description** is a user set parameter that succinctly describes the job. Limbs of the tree separate Cartesian PCA (**cPCA**), distance-pair PCA (**dpPCA**), and mode visualization analysis (**VIZ**) when present. Each of these in turn contains limbs for **Q** (**COV**), **R** (**CORR**), and **P (PCORR)** compartmentalization. Each PCA directory contains 3 subdirectories for the

subspace analysis (**SSA**). All output file names include the **number of residues or residue pairs** in the selected subset for reference, plus a description of the file contents.

Current Limitations:

Initial input of the protein trajectory must be done using PDB files that are expected to conform to the standard format, or a matrix of PDB coordinates containing the alpha carbon atomic positions only (see below for a description of this file). Only carbon-alpha atomic positions are used to create the coordinates matrix for essential dynamic analysis.

Each PDB file must have the exact same number of residues. The matrix of alpha carbon coordinates is determined from the first PDB file read. If other files in the working directory do not match exactly, then the array sizes will not match and the program will crash. *IF JED crashes during the reading of PDB files, this is probably the reason*.

While JED can process a PDB file with missing residues and various numbering schemes, it **cannot** interpret files that have alternate conformations within a given frame based on fractional **occupancy** values. Only a single conformation per frame is allowed. Note that the original residue coordinates in the PDB files are mapped to the rows of the coordinates matrix. A user should preprocess all PDB files and verify that they are error free and do not have ambiguities.

Note: JED reads all PDB files within a specified directory. Separate trajectories should be kept in different directories.

III. Overview of Using JED

A **Preliminary Run** must be performed to generate the JED formatted coordinate matrix file for all the alpha carbons in the PDB files. This makes subsequent subset analyses much faster to perform. It also serves to guarantee that the specified residues for subset selection are correctly represented in matrix form. After this initialization step, the PDB files can be deleted or archived, with the exception of the reference PDB file. The reference PDB is needed to make movies. Once the coordinate matrix is created, it should be used for all subsequent analyses, using different residue subsets and different job parameters.

The name of the coordinate file matrix produced from the PDB files is: `"original_PDB_coordinates.txt"`
The matrix packing is as follows:
Rows are coordinate variables and columns are frames.
For N residues, there are 3N rows: N x-coordinates, N y-coordinates, and N-z coordinates, stacked in that order.

- **The file to use in all subsequent JED analyses is the original_PDB_coordinates matrix.**

  This matrix contains all the residues in the PDB files and thus can be used for any subset of those residues. When a subset is chosen, a new correspondence set is generated and a new transformation is done to optimize the alignment of the structures. This removes overall translation and rotation for each subset chosen.

In subsequent analyses, it is critical that no residues are requested that do not exist in the PDB files! JED maps the specified residue list to an internal list that is aligned to the rows of the coordinates matrix. JED generates a residue list file for all residues it finds in the PDB files that it reads. Therefore, this residue-list file should be edited with care.

Note: The most critical step when using JED is in the creation of the input file. The input file must have the correct format (shown in examples below) and the entries must be accurate with proper ordering. If either of these conditions is violated, the program will crash, or worse, the results could be corrupt. To avoid producing un-intended results, JED provides error-handling feedback during most crashes so that the problems can be understood and addressed.

Common Causes for JED to Crash

- If **any specified directory** cannot be found or if **any specified file** cannot be found, JED will crash.
- If unexpected format is found in **any** of the input files, JED will crash.

The JED driver programs employ many consistency error checks during the reading of the input files and the execution of the program. There are checks to validate the number formats of numeric data, and to ensure enough parameters were specified for a particular job. There are checks to ensure that the input files have the correct format/number of columns, and to ensure the number of modes requested does not exceed the actual number of modes available. JED also verifies that directories and files exist before performing any analysis. In many cases, missing or problematic parameter settings are set to a default value. The developers have attempted to provide meaningful information when the program crashes to facilitate making the necessary corrections. The specified input file is echoed to ***standard out***, as well as assignment of parameters. Error messages are directed to ***standard error***. In the case that a Java runtime exception is thrown, a stack trace will be sent to standard error. Please refer to the **Appendix** for creating properly formatted input files.

IV. Using JED DRIVER:

A. The Preliminary Run

i. Run Command:

```
java -jar -d 64 JED_Driver.jar "/path/JED_Driver.txt"
```

The PDB files (including the PDB reference file) must be in the working directory.
JED input file may be in the working directory.

This pre-processing step will read all PDB files in the working directory, but will perform **no PCA.**
The purpose of this is to generate the matrix of coordinates for performing subset analyses efficiently.

ii. Root Output Files:

These are written to the **root** of the JED Results directory tree:
`/working/directory/JED_Results_Description/`

JED LOG providing a summary of the job parameters and results:

`JED_Log.txt`

PDB READ LOG listing all the PDB files read, in the order they were read:

`PDB_READ_Log.txt`

coordinates matrix from all the alpha carbon coordinates in the PDB files:

`original_PDB_coordinates.txt`

transformed coordinates matrix, which aligns all the frames to the reference frame :

`ss_$num_res_transformed_PDB_coordinates.txt`

list of all residues found in the PDB files for subsequent editing and use:

`All_PDB_Residues_JED.txt (for Single Chain PDBs)`

`All_PDB_Residues_Multi_JED.txt (for Multi Chain PDBs)`

original and transformed conformation RMSDs:

`ss_$num_res_original_conformation_rmsds.txt`

`ss_$num_res_conformation_rmsds.txt`

residue RMSDs (RMSF):

`ss_$num_res_residue_rmsd.txt`

edited PDB file containing all the residues with the RMSF replacing B-factors:

`ss_$num_res_edidited.PDB`

variables z-score matrix for all the alpha carbon coordinates in the PDB files:

```
ss_$num_res_Variable_Z_scores.txt
```

iii. JED Driver Input File Format: The Preliminary Run

```
-------------------------------------------------------------------------------------------
1     $multi
$working_directory
$description    $reference_PDB_file.pdb
-------------------------------------------------------------------------------------------
```

Notes: This is a **whitespace** separated file with **6 lines**.

Line 1 Field 1 specifies **read flag**, whether to **read PDB files** (0 or 1) → 1 = yes

Field 2: specifies **multi flag**, if the PDB files are **Multi Chain** (0 or 1)

Line 2 Field 1: specifies the **working directory** (String)

Line 3 Field 1: specifies the **description** (String) for the requested job

Field 2: specifies the **reference PDB** (String) for the requested job

----

Key Points:

- The **Read** flag **MUST** be set to **1** to perform the pre-processing runs
  - When the **Read** flag is set to **1**, all PCAs are turned off
- The **Multi** flag must be set to **0** for Single Chain PDBs with no Chain IDs
- The **Multi** flag must be set to **1** or "multi" for Multi Chain PDBs with Chain IDs
  - **Multi Chain PDBs must have unique chain identifiers for every chain**
  - **Missing chain identifiers will cause JED to crash**
- **The file to use in all subsequent JED analyses is the original_PDB_coordinates matrix**

B. Debugging Crashes Part I:

Things that will generally make your life miserable…

i. Simple mistakes:

Are the **Read** and **Multi** flags set correctly?
Does the path to the input file exist?
Does the input file exist in the proper location?
Does the input-file start on the first line?
Is the **number format** correct? (20.0 will NOT parse as an integer)
Did you forget a parameter declaration?
Does the working directory string end in "**/**" for Linux or "****" for Windows?
Does the working directory exist?
Does the working directory contain PDB files of different sizes?
Does the working directory contain the reference PDB file?
Does the reference PDB file exist?
Does the reference PDB file correspond to the trajectory?

ii. **Subtle mistakes:**

The directory contains PDB files in non-standard format.
The directory contains PDB files with fractional occupancy data.

The directory contains PDB files with 2 or more chains, but no chain IDs.
The directory contains PDB files with missing chain IDs.

Problem: If the PDB file names are sorted in a different order than how they were generated, then the conformation RMSD results will not reflect what actually occurred in the simulation. **Fix:** Naming the PDB files sequentially by padding the numbers with leading zeros will ensure proper sorting to prevent this problem caused by the operating system.

Problem: If the conformation RMSD is very different from what you expect, you may be using PDB files that contain occupancy information. JED does not use that information. Your results will not be accurate. **Fix:** Always perform error checking on your PDB files before using JED.

Problem: Trajectories from pooled data do not track original trajectories when they were individually analyzed. **Fix:** If you pool data together, make sure the combined matrix is constructed in the order you think it is, and the reference structure is the one you think it is. If done without error, you can always parse the output files by the same divisions to obtain information about each component trajectory, which can be colored differently and/or plotted separately, etc.

Problem: When making comparative analysis using cPCA across different proteins the covariance matrices look very different even though expectations are they should be similar. **Fix:** Do not use different reference structures when making comparisons between different trajectories. Always use the same reference structure for all cPCA procedures in order to directly compare the data.

C. Performing Only cPCA

i. Run command:

```
java -jar -d64 JED_Driver.jar “/path/JED_Driver.txt”
```

The working directory must contain: The coordinates matrix, the PDB reference file, and the Cartesian residue list.

The purpose of this type of run is to perform Essential Dynamics using cPCA based on **Q**, **R,** and **P**. The user specifies the subset of interest for the analysis, which may be the entire protein or a sub-region, which can be non-contiguous, by providing a residue list file. This task is simplified since JED has already created a list of all the residues in the protein. The user can simply edit this file. Keeping a copy of the original is usually best practice. The cPCA results are written to the sub-directory "cPCA" and the visualization of the top modes (when selected) are written to the subdirectory "VIZ". The directory cPCA has sub directories for the **Q, R** and **P** analysis, as does the VIZ directory.

ii. Root Output Files:

These are written to the **root** of the JED Results directory tree:
`/working/directory/JED_Results_Description/`

JED LOG providing a summary of the job parameters and results:

`JED_Log.txt`

iii. cPCA Output Files:

These are written to the **/cPCA subdirectory** of the JED Results directory tree:
`/working/directory/JED_Results_Description/cPCA/`

subset transformed coordinates matrix:

ss_**$num**_res_transformed_PDB_coordinates.txt

original and transformed conformation RMSDs:

ss_**$num**_res_original_conformation_rmsds.txt

ss_**$num**_res_conformation_rmsds.txt

residue RMSDs (RMSF):

ss_$num_res_residue_rmsd.txt

subset edited PDB file with the RMSF replacing B-factors:

ss_**$num**_res_edited.PDB

subset coordinates Z-Score matrix:

ss_**$num**_res_coordinate_Z_scores.txt

list of frames removed, based on the **percent** parameter:

ss_**$num**_res_ Removed_Conformation_Outliers.txt

trimmed transformed coordinate matrix:

ss_**$num**_res_ trimmed_$percent_percent_PDB_coordinates_COLS.txt

list of coordinate variables adjusted, based on the **z-cutoff** parameter:

ss_**$num**_res_ adjustments_per_variable.txt

adjusted transformed coordinate matrix:

ss_**$Z**_threshold_**$z**-cutoff_adjusted_PDB_coordinates_ROWS.txt

centroids (means) of the variables:

ss_**$num**_res_centroids_of_variables.txt

standard deviations of the variables:

ss_**$num**_res_std_devs_of_centered_variables.txt

displacement vectors:

ss_**$num**_res_delta_vectors.txt

- **The Q output files are written to the /COV subdirectory of /cPCA (shown below)**

/working/directory/JED_Results_Description/cPCA/COV/

- **The R output files are written to the /CORR subdirectory of /cPCA**

/working/directory/JED_Results_Description/cPCA/CORR/

- **The P output files are written to the /PCORR subdirectory of /cPCA**

/working/directory/JED_Results_Description/cPCA/PCORR/

covariance matrix:

ss_**$num_res**_covariance_matrix.txt

reduced covariance matrix:

ss_**$num_res**_reduced_covariance_matrix.txt

inverse covariance matrix:

ss_**$num_res**_inverse_covariance_matrix.txt

eigenvalues:

ss_**$num_res**_eigenvalues_COV.txt

top eigenvalues:

ss_**$num_res**_top_**$num**_of_cart_modes_eigenvalues_COV.txt

top eigenvectors:

ss_**$num_res**_top_**$num**_of_cart_modes_eigenvectors_COV.txt

top pca modes and top weighted pca modes:

ss_**$num_res**_top_**$num**_of_cart_modes_pca_modes_COV.txt
ss_**$num_res**_top_**$num**_of_cart_modes_weighted_pca_modes_COV.txt

top square pca modes and top weighted square pca modes:

ss_**$num_res**_top_**$num**_of_cart_modes_square_pca_modes_COV.txt
ss_**$num_res**_top_**$num**_of_cart_modes_weighted_square_pca_modes_COV.txt

top PCs and top weighted PCs:

ss_**$num_res**_top_**$num**_of_cart_modes_PCs_COV.txt
ss_**$num_res**_top_**$num**_of_cart_modes_weighted_PCs_COV.txt

top PCs and top weighted PCs:

ss_**$num_res**_top_**$num**_of_cart_modes_normed_PCs_COV.txt
ss_**$num_res**_top_**$num**_of_cart_modes_weighted_normed_PCs_COV.txt

iv. SSA Output Files:

These are written to the **/SSA subdirectory** of /cPCA:
/working/directory/JED_Results_Description/cPCA/SSA/

- **The CORR versus PCORR output files are written to the /CORR_VS_PCORR subdirectory of /SSA (shown below)**

/working/directory/JED_Results_Description/cPCA/SSA/CORR_vs_PCORR/

- **The CORR versus COV output files are written to the /CORR_VS_COV subdirectory of /SSA**

/working/directory/JED_Results_Description/cPCA/SSA/CORR_vs_COV/

- **The COV versus PCORR output files are written to the /COV_VS_PCORR subdirectory of /SSA**

/working/directory/JED_Results_Description/cPCA/SSA/COV_vs_PCORR/

The Fast SSA Iterated Log:

JED_FSSA_Iterated_log.txt

The SSA Log:

JED_SSA_dim_$top_num_cart_modes_log.txt

The Random SSA Log

JED_Random_SSA_log.txt

There are additional files in the /SSA/CORR_vs_PCORR directory that contain the results reported in the log files:

RMSIPs

PAs

COs

Cosine Products

Vectorial Sum of Angles

v. JED Driver Input File Format: Only cPCA

```
----------------------------------------------------------------------------------
0     $multi
$working_directory/
$description          $reference_PDB_file.pdb
$percent     $z_cutoff
$num_cPCA_modes     0     0
residues.txt
original_PDB_Coordinates.txt
----------------------------------------------------------------------------------
```

Notes: This is a **whitespace** delineated file with **7 lines**.

Line 1 Field 1 specifies **read flag**, whether to **read PDB files** (0 or 1) → **0 = no**

Field 2: specifies **multi flag**, if the PDB files are **Multi Chain** (0 or 1)

Line 2 Field 1 specifies the **working directory** (String)

Line 3 Field 1 specifies the job **description** (String)

Field 2 specifies the **reference PDB** (String)

Line 4 Field 1 specifies the **percent** (double)

Field 2 specifies the **z_cutoff** (double)

Line 5 Field 1 specifies the **number of cPCA modes** (integer)

Field 2: specifies the **number of dpPCA modes** (integer) → **0 = none**

Field 3: specifies the **number of Cartesian modes to Visualize**(integer) → **0 = none**

Line 6 Field 1 specifies the **Cartesian residue list** (String)

Line 7 Field1 specifies the **coordinate matrix** (String)

Key Points:

- The **Read** flag **MUST** be set to **0**
  - When the **Read** flag is set to **1**, all PCAs are turned off
- The **Multi** flag must be set to **0** for Single Chain PDBs with no Chain IDs
- The **Multi** flag must be set to **1** or "multi" for Multi Chain PDBs with Chain IDs
  - **Multi Chain PDBs must have unique chain identifiers for every chain**
  - **Missing chain identifiers will cause JED to crash**
- **The number of cPCA modes must NOT be 0.**
- **There must be a Cartesian residue list specified.**
- The number of dpPCA modes must be zero.
- There must not be a Distance or Distance Pair residue list specified.
- If the subset you have chosen contains N residues, then you must not request more than 3N modes.
- If you request N cPCA modes then you can only visualize up to N modes.

➢ **If number of cPCA modes > 0, then there must be a Cartesian residue list file!**

vi. <u>Residue List Format for cPCA</u>

The easiest way to create the residue list for cPCA is to edit the JED generated file that lists all residues found in the PDB files, in the proper format:

a. **Single Chain PDBs: "*All_PDB_Residues_JED.txt*"**

   ONE column of integers: residue numbers

b. **Multi Chin PDBs: "*All_PDB_Residues_Multi_JED.txt*"**

   TWO columns: Column 1 Strings, Column 2 integers (tab separated): Chain IDs, residue numbers

Note that all entries in the residue list are checked against the reference PDB file.

If a requested residue cannot be found in the reference file, then JED will crash with an error message stating that a requested residue could not be found.

D. Performing Only dpPCA

i. <u>Run command:</u>

```
java -jar -d64 JED_Driver.jar "/path/JED_Driver.txt"
```

The working directory <u>must</u> contain: The coordinates matrix, the PDB reference file, and the residue list.
JED input file <u>may</u> be in the working directory.

The purpose of this type of run is to perform Essential Dynamics using dpPCA based on **Q** , **R** and **P** models. The user specifies the set of **residue pairs** of interest for the analysis, by providing a *residue pair list file*. This file has two columns for Single Chain PDBs in which the pairs of interest are listed. However, for Multi Chain PDBs, the file has four columns, the first two for the chain ID and residue number of residue one, and the third and fourth columns for the chain ID and residue number of the second residue. The dpPCA results are written to the sub-directory "dpPCA". Note that for dpPCA no transform is needed since internal distances are used for coordinates and no visualization can be done in JED for the distance modes. The eigenvectors from dpPCA are easy to interpret as their components directly corresponding to the extension or compression of the distance pairs specified. The directory dpPCA has sub directories for the **Q** , **R** and **P** analysis, as well as for the subspace analysis (parallel to the cPCA method). Rationally selected distance pairs can be considered to investigate experimental findings in critical areas like binding pockets or clefts.

<u>Note</u>: Unfortunately, the dpPCA results cannot be visualized as no simple mapping can be made to the residues.

ii. <u>Root Output Files:</u>

These are written to the **root** of the JED Results directory tree:
`/working/directory/JED_Results_Description/`

JED LOG providing a summary of the job parameters and results:

`JED_Log.txt`

iii. <u>dpPCA Output Files:</u>

These are written to the **/dpPCA subdirectory** of the JED Results directory tree:

`/working/directory/JED_Results_Description/dpPCA/`

variables Z-Score matrix:

`ss_$num_res_pairs_Distance_Pairs_distance_Z_scores.txt`

list of distance-pair variables adjusted, based on the **z-cutoff** parameter:

`ss_$num_res_pairs_Distance_Pairs_outliers_per_variable.txt`

distance residue stats from all the distance pairs in the **pair list**:

`ss_$num_res_pairs_Distance_Pairs_distance_residue_stats.txt`

distance Z-Score matrix from all the distance pairs in the **pair list**:

ss_**$num_res_pairs**_Distance_Pairs_distance_Z_scores.txt

distances matrix from all the distance pairs in the **pair list**:

ss_**$num_res_pairs**_Distance_Pairs_distances.txt

centroids (means) of the variables:

ss_**$num_res_pairs**_Distance_Pairs_centroids_of_variables.txt

standard deviations of the variables:

ss_**$num_res_pairs**_Distance_Pairs_std_devs_of_centered_variables.txt

displacement vectors:

ss_**$num_res_pairs**_Distance_Pairs_delta_vectors.txt

- **The Q output files are written to the /COV subdirectory of /dpPCA** (Shown below)

/working/directory/JED_Results_Description/dpPCA/COV/

- **The R output files are written to the /CORR subdirectory of /dpPCA**

/working/directory/JED_Results_Description/dpPCA/CORR/

- **The P output files are written to the /PCORR subdirectory of /dpPCA**

/working/directory/JED_Results_Description/dpPCA/PCORR/

covariance matrix:

ss_**$num_res_pairs**_Distance_Pairs_distance_pair_covariance_matrix.txt

inverse covariance matrix:

ss_**$num_res_pairs**_Distance_Pairs_distance_pair_inverse_covariance_matrix.txt

eigenvalues:

ss_**$num_res_pairs**_Distance_Pairs_eigenvalues_COV.txt

top eigenvalues:

ss_**$num_res_pairs**_Distance_Pairs_top_**$num_of_dist_modes**_eigenvalues_COV.txt

top eigenvectors:

ss_**$num_res_pairs**_Distance_Pairs_top_**$num_of_dist_modes**_eigenvectors_COV.txt

top pca modes and top weighted pca modes:

ss_**$num_res_pairs**_Distance_Pairs_top_**$num_of_dist_modes**_pca_modes_COV.txt
ss_**$num_res_pairs**_Distance_Pairs_top_**$num_of_dist_modes**_weighted_pca_modes_COV.txt

top square pca modes and top weighted square pca modes:

ss_**$num_res_pairs**_Distance_Pairs_top_**$num_of_dist_modes**_square_pca_modes_COV.txt
ss_**$num_res_pairs**_Distance_Pairs_top_**$num_of_dist_modes**_weighted_square_pca_modes_COV.txt

top DVPs and top weighted DVPs:

ss_**$num_res_pairs**_Distance_Pairs_top_**$num_of_dist_modes**_DVPs_COV.txt
ss_**$num_res_pairs**_Distance_Pairs_top_**$num_of_dist_modes**_weighted_DVPs_COV.txt

top normed DVPs and top weighted normed DVPs:

ss_**$num_res_pairs**_Distance_Pairs_top_**$num_of_dist_modes**_normed_DVPs_COV.txt
ss_**$num_res_pairs**_Distance_Pairs_top_**$num_of_dist_modes**_weighted_normed_DVPs_COV.txt

iv. <u>SSA Output Files:</u> Same as for cPCA

These are written to the **/SSA subdirectory** of /dpPCA:
`/working/directory/JED_Results_Description/dpPCA/SSA/`

v. <u>JED Driver Input File Format: Only dpPCA</u>

```
-------------------------------------------------------------------------------------
0     $multi
$working_directory/
$description          $reference_PDB_file.pdb
$percent     $z_cutoff
0      $num_dpPCA_modes     0
residue_pairs.txt
original_PDB_Coordinates.txt
-------------------------------------------------------------------------------------
```

<u>Notes</u>: This is a **whitespace** delineated file with **7 lines**.

Line 1 Field 1 specifies **read flag**, whether to **read PDB files** (0 or 1) → **0 = no**
Field 2: specifies **multi flag**, if the PDB files are **Multi Chain** (0 or 1)
Line 2 Field 1 specifies the **working directory** (String)
Line 3 Field 1 specifies the job **description** (String)
Field 2 specifies the **reference PDB** (String)
Line 4 Field 1 specifies the **percent** (double)
Field 2 specifies the **z_cutoff** (double)
Line 5 Field 1 specifies the **number of cPCA modes** (integer) → **0 = none**
Field 2: specifies the **number of dpPCA modes** (integer) →10 = top 10 modes
Field 3: specifies the **number of Cartesian modes to Visualize** (integer) → **0 = none**
Line 6 Field 1 specifies the **Distance Pairs residue list** (String)
Line 7 Field1 specifies the **coordinate matrix** (String)

<u>Key Points</u>:

- The **Read** flag **<u>MUST</u>** be set to **0**
  - When the **Read** flag is set to **1**, all PCAs are turned off
- The **Multi** flag must be set to **0** for Single Chain PDBs with no Chain IDs
- The **Multi** flag must be set to **1** or "multi" for Multi Chain PDBs with Chain IDs
  - **Multi Chain PDBs <u>must</u> have unique chain identifiers for every chain**
  - **Missing chain identifiers will cause JED to crash**
- The number of cPCA modes must be 0.
- There must be no Cartesian residue list specified.
- **The number of dpPCA modes must NOT be zero.**
- **There MUST be a Distance Pair residue list specified.**
- If the subset you have chosen contains ***N*** pairs, then you must NOT request more than ***N*** modes.

- **If number of dpPCA modes > 0, then there must be a Distance Pair Residue list file!**

vi. **Residue List Format for dpPCA**

Residue pairs are specified one to a line in the residue pair list file:

a. **Single Chain PDBs: Two columns of integers, tab separated:**

residue number1 residue number2

b. **Multi Chin PDBs: Four columns of strings and integers, tab separated:**

ChainID1 Res_Number1 Chain ID2 Res_Number2

Note that all entries in the residue list are checked against the reference PDB file.

If a requested residue cannot be found in the reference file, then JED will crash with an error message stating that a requested residue could not be found.

E. **Debugging Crashes Part II:**

Things that will generally make your life miserable…

i. **Simple mistakes:**

Did you set the **Read** and **Multi** flags correctly?
Are you requesting to read PDBs when you are doing an analytical run?
Did you request cPCA but not specify a Cartesian residue list?
Did you request dpPCA but not specify a Distance Residue Pair List?
Did you set the number of modes appropriately?
Are you requesting residues that are not in the reference PDB?

ii. **Subtle mistakes:**

Did you request more PCA modes than actually exist?

For example (cPCA):

If your Cartesian subset contains 12 residues and you ask for 50 modes, then you are going get error messages: Because there are only 36 Cartesian modes in total.

For example (dpPCA):

If your Distance Pairs List contains 5 pairs and you request 10 modes, then you are going to get error messages:

Because there are only 5 distance-pair modes in total.

In the above cases, JED will attempt to reset the offending value.

If your trajectory has not equilibrated, then you must address the problem of outliers. If you do not, then the covariance matrix will be highly ill-conditioned and may cause the eigenvalue decomposition to fail. You can check the variables in statistics packages that compute the Kaiser-Myer-Olkin (KMO) statistic as well as the Measure of Sampling Adequacy (MSA) for each coordinate variable to critically assess your data. If it is not well suited for PCA, you can condition the variables by setting the z-cutoff in JED between 2.0 and 3.0 when running your jobs. This type of conditioning is by far not very sophisticated, but it has the effect of lowering the condition numbers of Q and R as well as un-dilating the high and low regions of the eigenspectrum. In particular, it does not alter the ordinality of the eigenvalues, but does correct the distortion that arises from under sampling when trying to estimate the population covariance matrix from a poor sample covariance matrix.

Examination of the correlation matrix, in conjunction with the partial correlation matrix, can provide insight into the amount of correlation between the variables, and how many variables are conditionally independent.

Note: The KMO and MSA are determined by using the correlations and partial correlations.

F. Visualizing Cartesian Modes as an Animation

To visualize cPCA modes as an animation, you must be running a job with cPCA selected. To generate the output files, you need to **set the number of modes VIZ > 0 (**and **≤ number of available cPCA modes)**, and **set the mode amplitude**. If the mode amplitude is not set, the **default value of 1.5** will be applied. A mode amplitude of 1.5 usually provides good looking movies, however, movie characteristics are somewhat subjective depending on the system of interest and what is being shown. Adjusting this mode amplitude should be done as trial and error after JED completes the calculation. The output from this job creates movies for vibrations of the top modes chosen for visualization for all three models. JED perturbs the reference structure within the selected sub-region based on the eigenvectors. Using a sine function, the dynamics show as harmonic vibrations. To capture one cycle, 20 structures (PDBs) are used as frames for these movies, which repeat indefinitely using PyMol™ scripts to show periodic motion.

Note: Setting the modes VIZ flag to zero turns off the visualization feature.

Additionally, JED constructs an Essential Modes Visualization that is comprised of a superposition of the top chosen modes or the top 5, which ever is less. The process is similar to how individual modes are handled, with frequencies increasing and amplitude decreasing as mode number increases. When the ratios of eigenvalues are not whole numbers, the PyMol™ script that cycles indefinitely will show a discontinuity because higher frequencies will not be multiples of the lowest frequency. Because relative amplitudes of higher modes decreases rapidly, contributions to essential motion from higher frequency mode can be windowed to look at different time-scales.

These files will be located in the /VIZ subdirectory of the root of the JED results tree:
`/working/directory/JED_Results_Description/VIZ/`

The **Q** results will be in the subdirectory /COV

The **R** results will be in the subdirectory /CORR.

The **P** results will be in the subdirectory /PCORR.

G. Performing Multiple PCAs

The working directory must contain: The coordinates matrix, the PDB reference file, and the residue lists.

JED is capable of doing cPCA (with or without visualization), and dpPCA simultaneously.
All outputs are delivered as discussed for the individual components.

JED expects the input file to follow the following format regarding the order of the residue lists:

If cPCA then Cartesian Residue List
If dpPCA then Distance Pair Residue List

An important advantage of JED is that it is highly configurable to perform many types of Essential Dynamics analysis concurrently. Combined with cluster resources or just using the batch feature (discussed in the next section) allows a user to process a great deal of data efficiently.

i. JED Driver Input File Format: Combined cPCA, dpPCA, VIZ

```
-------------------------------------------------------------------------------------------
0     $multi
$working_directory/
$description         $reference_PDB_file.pdb
0.00     3.00
$num_cPCA_modes      $num_dpPCA_modes     $num_viz_modes     $mode_amplitude
residues_cartesian.txt
residue_pairs.txt
original_PDB_Coordinates.txt
-------------------------------------------------------------------------------------------
```

Key Points:

- The **Read** flag **MUST** be set to **0**
- The **Multi** flag must be set to **0** for Single Chain PDBs with no Chain IDs
- The **Multi** flag must be set to **1** or "multi" for Multi Chain PDBs with Chain IDs
- If the number of cPCA modes > 0, then there must be a Cartesian residue list specified
- If the number of dpPCA modes > 0, then there must be a Distance Pair residue list specified

V. USING JED BATCH DRIVER

The batch version is identical to the non-batch version with the exception of the format of the input file.

A. JED Batch Driver Input File Format: The Preliminary Run

```
-------------------------------------------------------------------------------------------
$num_of_jobs
1     $multi
***************************************************************************
$working/directory/job1/
$description1              $reference_PDB_file1.pdb
***************************************************************************
$working/directory/job2/
$description2              $reference_PDB_file2.pdb
***************************************************************************
-------------------------------------------------------------------------------------------
```

Notes: This is a whitespace delineated file.

Line 1 Field 1 specifies the **$num_of_jobs** (integer) for the batch.

Line 2 Field 1 specifies the **read flag**, whether to **read PDB files** (0 or 1) → 1 = yes

Field 2 specifies the **multi flag**, if the PDB files are **Multi** Chain (0 or 1) → 0 = no

Line 3 is a **separator line** between the batch parameters and the job parameters *************************

Line 4 Field 1 specifies the **working directory** (String) for job one

Line 5 Field 1 specifies the job **description** (String) for job one

Field 2 specifies the **reference PDB** (String) for job one

Line 6 is a **separator line** between sets of job parameters ***

Line 7 Field 1 specifies the **working directory** (String) for job one

Line 8 Field 1 specifies the job **description** (String) for job two

Field 2 specifies the **reference PDB** (String) for job two

Line 9 is a **separator line** between sets of job parameters ***

Key Points:

- **Be sure that the number of jobs matches the number of job inputs.**
- The **Read** flag **MUST** be set to **1** or **"read"**
- The **Multi** flag must be set to **0** for Single Chain PDBs with no Chain IDs
- The **Multi** flag must be set to **1** or "multi" for Multi Chain PDBs with Chain IDs
- **Make sure to use separator lines after the batch parameters, between jobs, and after the last job.**

B. JED Batch Driver Input File Format: Only cPCA

```
----------------------------------------------------------------------------------------
2
0     0
0.01     3.00
20    0     0
****************************************************************************************
$working/directory1/
$description1         $reference_PDB_file1.pdb
$residues1.txt
original_PDB_Coordinates.txt
****************************************************************************************
$working/directory2/
$description2         $reference_PDB_file2.pdb
$residues2.txt
original_PDB_Coordinates.txt
****************************************************************************************
----------------------------------------------------------------------------------------
```

Notes:

Line 1 Field 1 specifies the **$num_of_jobs** (integer) for the batch → **2**

Line 2 Field 1 specifies the **read flag**, whether to **read PDB files** (0 or 1) → **0 = no**

Field 2 specifies the **multi flag**, if the PDB files are **Multi** Chain (0 or 1) → **0 = no**

Line 3 Field 1 specifies the **percent** (double) of frames to remove from the data: **0.01 → 1%**

Field 2 specifies the **z-cutoff** (double) for adjusting outliers: **3.00 → values beyond |3.0|**

Line 4 Field 1 specifies the **number of cPCA** modes (integer) → **20 = top 20 modes**

Field 2 specifies the **number of dpPCA** modes (integer) → **0 = none**

Field 3 specifies the **number of Cartesian modes to Visualize** (integer) → **0 = none**

Line 5 is a **separator line** between the batch parameters and the job parameters ************************

Line 6 Field 1 specifies the **working directory** (String) for job one

Line 7 Field 1 specifies the job **description** (String) for job one

Field 2 specifies the **reference PDB** (String) for job one

Line 8 Field 1 specifies the **residue list** (String) for job one

Line 9 Field 1 specifies the **coordinate matrix** (String) for job one

Line 10 is a **separator line** between sets of job parameters **

Line 11 Field 1 specifies the **working directory** (String) for job two

Line 12 Field 1 specifies the job **description** (String) for job two

Field 2 specifies the **reference PDB** (String) for job two

Line 13 Field 1 specifies the **residue list** (String) for job two

Line 14 Field 1 specifies the **coordinate matrix** (String) for job two

Line 15 is a **separator line** between sets of job parameters **

C. JED Batch Driver Input File Format: Only dpPCA

```
--------------------------------------------------------------------------------
2
0     1
0.01     3.00
0     3     0
*******************************************************************************
$working/directory1/
$description1          $reference_PDB_file1.pdb
$residues_pairs1.txt
original_PDB_Coordinates1.txt
*******************************************************************************
$working/directory2/
$description2          $reference_PDB_file2.pdb
$residues_pairs2.txt
original_PDB_Coordinates2.txt
*******************************************************************************
--------------------------------------------------------------------------------
```

Notes:

Line 1 Field 1 specifies the **$num_of_jobs** (integer) for the batch

Line 2 Field 1 specifies the **read flag**, whether to **read PDB files** (0 or 1) → **0 = no**

Field 2 specifies the **multi flag**, if the PDB files are **Multi** Chain (0 or 1) → **1 = yes**

Line 3 Field 1 specifies the **percent** (double) of frames to remove from the data: **0.01 → 1%**

Field 2 specifies the **z-cutoff** (double) for adjusting outliers: **3.00 → values beyond |3.0|**

Line 4 Field 1 specifies the **number of cPCA** modes (integer) → **0 = none**

Field 2 specifies the **number of dpPCA** modes (integer) → **3 = top 3 modes**

Field 3 specifies the **number of Cartesian modes to Visualize** (integer) → **0 = none**

Line 5 is a **separator line** between the batch parameters and the job parameters ************************

Line 6 Field 1 specifies the **working directory** (String) for job one

Line 7 Field 1 specifies the job **description** (String) for job one

Field 2 specifies the **reference PDB** (String) for job one

Line 8 Field 1 specifies the **residue pair list** (String) for job one

Line 9 Field 1 specifies the **coordinate matrix** (String) for job one

Line 10 is a **separator line** between sets of job parameters **

Line 11 Field 1 specifies the **working directory** (String) for job two

Line 12 Field 1 specifies the job **description** (String) for job two

Field 2 specifies the **reference PDB** (String) for job two

Line 13 Field 1 specifies the **residue pair list** (String) for job two

Line 14 Field 1 specifies the **coordinate matrix** (String) for job two

Line 15 is a **separator line** between sets of job parameters **

D. JED Batch Driver Input File Format: Combined cPCA, dpPCA, VIZ

```
-----------------------------------------------------------------------------------
$num_of_jobs
0     $multi
0.00     3.00
20    3     2     1.5
***********************************************************************************
$working/directory1/
$description1         $reference_PDB_file1.pdb
$residues1.txt
$residues_pairs1.txt
original_PDB_Coordinates.txt
***********************************************************************************
$working/directory2/
$description2         $reference_PDB_file2.pdb
$residues2.txt
$residues_pairs2.txt
original_PDB_Coordinates.txt
***********************************************************************************
-----------------------------------------------------------------------------------
```

Key Points:

- **Be sure that the number of jobs matches the number of job inputs**
- The **Read** flag **MUST** be set to **0**
- The **Multi** flag must be set to **0** for Single Chain PDBs with no Chain IDs
- The **Multi** flag must be set to **1** or "multi" for Multi Chain PDBs with Chain IDs
- Make sure to use **separator lines** after the batch parameters, between jobs, and after the last job
- Make sure to specify the residue lists in the **correct order**: **cPCA first, dpPCA second**
- Make sure to specify the desired **$mode_amplitude** if you request cPCA mode visualization

E. Debugging Batch Crashes:

Handling batch problems is more difficult than for single jobs. Any problem in any job can cause a crash.
Thus, it is good to track the standard out and errors streams to record the cause of any problems.
Also, you will know which jobs ran successfully so that you can edit the batch input file to finish the undone jobs.

i. Simple mistakes:

Are the **Read** and **Multi** flags set correctly?
Is the **number format** correct? (20.0 will NOT parse as an integer)
Do ALL the working directories, residue lists, and PDB reference files **exist**?
Do ALL the working directories end in "**/**" for Linux or "****" for Windows?

Does the working directory contain **ALL of the required files**? (2 Types of PCA = 2 residue list input files)

ii. More subtle mistakes:

Do any of the jobs have residue sets that will not allow the batch parameters to apply?
For example, too few residues for the number of modes specified.
Does the directory contain PDB files in **non-standard format** or with fractional **occupancy** data?
Does the reference PDB file correspond to the trajectory? (JED must map the ref pdb to the coords matrix)
Does the directory contain Multi Chain PDB files with missing chain IDs?

VI. Additional Types of Analysis

A. Pooling Data:

It is often useful to pool trajectory statistics. This can be done in JED by combining coordinate files and then performing the usual analysis. To combine the coordinate files, there is a utility program called **Pool_Driver.java** that will combine multiple matrices into one. Each matrix is appended to the last column of the preceding matrix. Of course, the number of rows in the coordinate files must match.

The matrices to combine are specified by an input file called **POOL.txt** that the user must construct correctly.

i. Run Command:

```
java -d64 Pool_Driver.java "/path/to/POOL.txt"
java -jar -d64 Pool_Driver.jar "/path/to/POOL.txt"
```

ii. Input File format:

LINE 1 specifies the number of jobs (integer)
LINE 2 is a separator line ***********************************
Then for each job you must specify the following:
The number of matrices to combine (integer)
The job description (String)
The output directory (string ending in "/" or "\\")
The path to each matrix (String)
A separator line at end of job declaration **

Sample "POOL.txt":

```
-----------------------------------------------------------------------------------------
2
*****************************************************************************************
3
description1
/output/directory1/
/path/to/first/coords/matrix/original_PDB_Coordinates.txt
/path/to/second/coords/matrix/original_PDB_Coordinates.txt
/path/to/third/coords/matrix/original_PDB_Coordinates.txt
*****************************************************************************************
4
description2
/output/directory2/
```

```
/path/to/first/coords/matrix/original_PDB_Coordinates.txt
/path/to/second/coords/matrix/original_PDB_Coordinates.txt
/path/to/third/coords/matrix/original_PDB_Coordinates.txt
/path/to/fourth/coords/matrix/original_PDB_Coordinates.txt
*************************************************************************************************
```

Notes:

This file specifies 2 jobs, with 3 matrices to combine for job 1, and 4 matrices to combine for job 2.
Be sure that each path and matrix file exists.

---

iii. Output File format:

The output is a single, augmented matrix with the same number of rows as the composite matrices and columns equal to the sum of all columns in the composite matrices.

The output file name is: **Pooled_Coordinates_Matrix_$description_$*number-of-input-matrices*.txt**

B. Subspace Analysis:

Once JED Driver has been run on multiple trajectories, as well as pooled trajectories, an analysis can be done to compare how similar the essential subspaces derived from those trajectories are to each other. JED contains a program called **Subspace_Analysis.java** along with 3 driver programs that perform those functions. The core program takes as input two matrices of eigenvectors derived from PCA (or NMA, ANM, etc.). **The matrices must have the same number of rows and columns, meaning the vectors being compared come from the same vector space having the same dimension**. For example, as part of an analysis you might choose to process 20 cPCA modes while examining 10 different experimental conditions, plus pooled data. As long as all subsets in the analysis are the same, then they all can be directly compared to one another. Note that modes from other methods (e.g. ENM or ANM) can also be compared if they have the same dimension, but care should be taken to identify which eigenvectors from different methods map into similar subspaces.

Like most of the JED programs, the subspace analysis program driver reads an input file called **SSA.txt** to obtain runtime information. This file must be constructed properly to perform the analysis correctly. The three driver programs are **SSA_Driver.java**, **FSSA_Driver.java**, and **FSSA_Iterated_Driver.java** and are different in how much analysis is requested. The SSA_Driver gives full outputs for non-iterated subspace comparison including both log files and individual flat files. The FSSA_Driver is a light-weight version with only RMSIP and PA output in the log files. The Iterated version performs a recursive variation of the above where all equidimensional subspaces are compared up to the size that was provided, for example, from 1 to 20 by step-size 1 for a 20 column input file.

i. Run Commands:

```
java -jar -d64 SSA_Driver.jar "/path/to/SSA.txt"
java -jar -d64 FSSA_Driver.jar "/path/to/SSA.txt"
java -jar -d64 FSSA_Iterated_Driver.jar "/path/to/SSA.txt"
```

ii. Input File format:

ALL three drivers use the same input file (only the outputs are different)

The format for SSA.txt is shown below:

LINE 1: Number_of_Jobs (integer)
LINE 2: Output_Directory (string ending in "/" or "\\")
LINE 3: Batch_Decription (string)
LINE 3: Separator Line **
THEN FOR EACH JOB:
Description (string)
$directory1 (string ending in "/" or "\\")** **$eigenvectors1 (string)
$directory2 (string ending in "/" or "\\")** **$eigenvectors2 (string)
Separator Line ***
Sample "SSA.txt":

```
----------------------------------------------------------------------------------------------------
5
/output/directory/
MV_PCA_Model_Test
****************************************************************************************************
MV1
/path/to/first/eigenvector/matrix/                 ss_946_top_20_eigenvectors_COV.txt
/path/to/second/eigenvector/matrix/                ss_946_top_20_eigenvectors_CORR.txt
****************************************************************************************************
MV2
/path/to/first/eigenvector/matrix/                 ss_946_top_20_eigenvectors_COV.txt
/path/to/second/eigenvector/matrix/                ss_946_top_20_eigenvectors_CORR.txt
****************************************************************************************************
MV3
/path/to/first/eigenvector/matrix/                 ss_946_top_20_eigenvectors_COV.txt
/path/to/second/eigenvector/matrix/                ss_946_top_20_eigenvectors_CORR.txt
****************************************************************************************************
MV4
/path/to/first/eigenvector/matrix/                 ss_946_top_20_eigenvectors_COV.txt
/path/to/second/eigenvector/matrix/                ss_946_top_20_eigenvectors_CORR.txt
****************************************************************************************************
MV5
/path/to/first/eigenvector/matrix/                 ss_946_top_20_eigenvectors_COV.txt
/path/to/second/eigenvector/matrix/                ss_946_top_20_eigenvectors_CORR.txt
****************************************************************************************************
----------------------------------------------------------------------------------------------------
```

C. Free Energy Surface:

JED contains a program called FES_Driver.java that takes two DVPs as order parameters (OP) to calculate free energy (FE) using a 2-D kernel density estimate (KDE) derived from Gaussian kernels. The output file has three columns: OP1, OP2, FE. This output can be used to plot a free energy surface with respect to the selected PCA modes. The FES can be done for both cPCA and dpPCA, and for any PCA model. The user specifies the OPs, the number of conformations to use, the offset into the points (which determines the points to use in the KDE), and the size of the 2D grid elements for making the KDE. All required information is specified in the input file FES.txt

i. Run Commands:

```
java -jar -d64 FES_Driver.jar "/path/to/FES.txt"
java -d64 FES_Driver.java "/path/to/FES.txt"
```

ii. Input File format:

The format for FES.txt is shown below:

LINE 1: Number_of_Jobs (integer)
LINE 2: Output_Directory (string ending in "/" or "\\")
LINE 3: Batch_Decription (string)
LINE 3: Separator Line **
THEN FOR EACH JOB:
Description (string)
$directory1 (string ending in "/" or "\\")
$delta_vectors (string)
$OP1 (int) $OP2 (int) $num_points (int) $offset (int) $cellsize (double)
Separator Line ***
<u>Sample "FES.txt":</u>

```
----------------------------------------------------------------------------------------------------
2
/output/directory/
Test_FES_Pooled_Data
******************************************************************************************************
Test_First_Half
/working/directory1/
ss_151_top_2_DVPs_CORR.txt
0     1     1001     0     0.00
******************************************************************************************************
Test_Second_Half
/working/directory1/
ss_151_top_2_DVPs_CORR.txt
0     1     1000     1000     0.00
******************************************************************************************************
----------------------------------------------------------------------------------------------------
```

<u>Notes</u>:
Recommended values for cellsize is [0.01, 0.05]
Set $size = 0 to determine automatically, using the Max (Range X, Range Y)/256

D. Essential Mode Visualization:

JED contains a program called VIZ_Driver.java that allows the user to view the superposition of a set of selected modes. A starting mode is specified as the leading edge of a window. The number of modes to visualize determines the size of that window. To enhance the log-coloring scheme, low and high thresholds can be adjusted for a percentage of frames to be set to minimum and maximum values, broadening the coloring of the inter-threshold range. The mode amplitude parameter determines the amount of displacement from equilibrium for visualizing the modes. This driver affords the user considerable control for the visualization process, including the number of frames to generate and the number of cycles to capture for the slowest mode. All required information is specified in the input file VIZ.txt

i. <u>Run Commands:</u>

```
java -jar -d64 VIZ_Driver.jar "/path/to/VIZ.txt"
java -d64 VIZ_Driver.java "/path/to/VIZ.txt"
```

ii. <u>Input File format:</u>

The format for VIZ.txt is shown below:

LINE 1: Number_of_Jobs (integer)
LINE 2: Separator Line **
THEN FOR EACH JOB:

$start_mode $num_modes_viz $mode_amplitutude $threshold_low $threshold_high $num_frames $num_cycles $do_individual $type

$ref_PDB

$eigenvalues

$eigenvectors

$sq_modes

$mode_maxes

$mode_mins

$out_dir (string ending in "/" or "\\")

Separator Line **

<u>Sample "VIZ.txt":</u>

```
-------------------------------------------------------------------------------
2
*************************************************************************************
1     5     3.0     .05     .05     100     5     0     PCORR
/path/to/ss_151_RMSF_edited.pdb
/path/to/ss_151_eigenvalues_PCORR.txt
/path/to/ss_151_top_20_eigenvectors_PCORR.txt
/path/to/ss_151_top_20_square_pca_modes_PCORR.txt
/path/to/ss_151_top_20_square_pca_mode_MAXES_PCORR.txt
/path/to/ss_151_top_20_square_pca_mode_MINS_PCORR.txt
/output/directory/VIZ_TEST_PCORR/
*************************************************************************************
1     5     3.0     .05     .05     100     5     0     CORR
/path/to/ss_151_RMSF_edited.pdb
/path/to/ss_151_eigenvalues_CORR.txt
/path/to/ss_151_top_20_eigenvectors_CORR.txt
/path/to/ss_151_top_20_square_pca_modes_CORR.txt
/path/to/ss_151_top_20_square_pca_mode_MAXES_CORR.txt
/path/to/ss_151_top_20_square_pca_mode_MINS_CORR.txt
/output/directory/VIZ_TEST_CORR/
*************************************************************************************
-------------------------------------------------------------------------------
```

iv. <u>Output File format:</u>

The output is a set of PDBs of size **$num_frames** and sets of twenty PDBs for the individual modes if requested by setting **$do_individual** to “1”. Also, PyMol™ scripts are generated to animate the sets of PDB files (.pml files).

APPENDIX

I. Input File Formats

A. JED_Driver.txt (Pre-Processing Runs)

```
-----------------------------------------------------------------------------------
$read          $multi
$working_directory
$description   $reference_PDB
-----------------------------------------------------------------------------------
```

B. JED_Driver.txt (Analytical Runs)

```
-----------------------------------------------------------------------------------
$read          $multi
$working_directory
$description   $reference_PDB
$percent       $z-cutoff
$num_cPCA_modes      $num_dpPCA_modes     $num_viz_modes     $mode_amplitude
$residues_cartesian                                                     (Needed for cPCA)
$residue_pairs.txt                                                      (Needed for dpPCA)
$PDB_Coordinates
-----------------------------------------------------------------------------------
```

C. JED_Batch_Driver.txt (Pre-Processing Runs)

```
-----------------------------------------------------------------------------------
$num_jobs
$read          $multi
****************************************************************************
$working_directory
$description   $reference_PDB
****************************************************************************
-----------------------------------------------------------------------------------
```

D. JED_Batch_Driver.txt (Analytical Runs)

```
--------------------------------------------------------------------------------
$num_jobs
$read          $multi
$percent       $z-cutoff
$num_cPCA_modes       $num_dpPCA_modes     $num_viz_modes     $mode_amplitude
********************************************************************************
$working_directory
$description   $reference_PDB
$residues_cartesian                                                   (Needed for cPCA)
$residue_pairs.txt                                                    (Needed for dpPCA)
$PDB_Coordinates
********************************************************************************
--------------------------------------------------------------------------------
```

E. Residue List file for cPCA: Single Chain PDBs

```
--------------------------------------------------------------------------------
1
2
3
7
8
9
10
23
24
25
--------------------------------------------------------------------------------
```

F. Residue List file for cPCA: Multi Chain PDBs

```
--------------------------------------------------------------------------------
A      1
A      2
A      3
A      7
A      8
A      9
A      10
B      1
B      2
B      3
B      4
B      5
--------------------------------------------------------------------------------
```

G. Residue Pair List file for dpPCA: Single Chain PDBs

```
--------------------------------------------------------------------------------
2             27
5             32
12            57
18            80
36            101
```

```
-------------------------------------------------------------------------------------
```

H. Residue Pair List file for dpPCA: Multi Chain PDBs

```
-------------------------------------------------------------------------------------
A             2             A             27
A             5             A             32
A             12            B             57
B             18            B             80
B             36            B             101
-------------------------------------------------------------------------------------
```

I. POOL.txt

```
J. -------------------------------------------------------------------------------------
$num_jobs --> (repeat job declaration $num_jobs times)
*****************************************************************************************
$num_of_matrices_to_combine
$description
$output_directory
$path_to_coords_matrix1 --> (repeat line $num_of_matrices_to_combine times)
*****************************************************************************************
-------------------------------------------------------------------------------------
```

K. SSA.txt

```
L. -------------------------------------------------------------------------------------
$num_jobs --> (repeat job declaration $num_jobs times)
$output_directory
$batch_description
*****************************************************************************************
$job_description
$path_to_first_eigenvector_file
$path_to_second_eigenvector_file
*****************************************************************************************
-------------------------------------------------------------------------------------
```

M. FES.txt

```
-------------------------------------------------------------------------------------
$num_jobs --> (repeat job declaration $num_jobs times)
$out_dir
$batch_description
****************************************************
$job_description
$directory
$delta_vectors
$op1     $op2     $num_points     $offset     $size
****************************************************
-------------------------------------------------------------------------------------
```

N. VIZ.txt

```
-------------------------------------------------------------------------------------
$num_jobs --> (repeat job declaration $num_jobs times)

*********************************************************************************************************
```

```
$start_mode   $num_modes_viz   $mode_amplitutude   $threshold_low   $threshold_high   $num_frames   $num_cycles   $do_individual   $type

$ref_PDB

$eigenvalues

$eigenvectors

$sq_modes

$mode_maxes

$mode_mins

$out_dir
*************************************************************************************************************
-------------------------------------------------------------------------------------------------------------
```

II. Output File Formats

A. Sample JED Log file: cPCA, dpPCA, VIZ

```
-------------------------------------------------------------------------------------------------
JED: Java Essential Dynamics version 1.0
Job Description: TEST
Working directory: C:\\Users\\Charles\\workspace\\JED_2.0\\JED_Test\\Multi\\
Output directory: C:\\Users\\Charles\\workspace\\JED_2.0\\JED_Test\\Multi\\JED_RESULTS_TEST\\
READ PDBs = false
MULTI CHAIN PDBs = true

The alpha carbon coordinates were obtained from coordinates matrix file: original_PDB_Coordinates.txt
The dimension of the coordinates matrix is = 2838 by 101
Total number of residues in matrix = 946
Total number of conformations in matrix = 101
Transformed PDB coordinates obtained by quaternion least-squares alignment to the reference structure.
PDB reference structure is: MVb_A_B_ATP.pdb

PERFORMING cPCA, Computing Top 5 modes.
Residue list for Cartesian subset:  residues.txt
Number of residues in Cartesian subset: 50
No samples were removed from the data
No coordinate outliers were adjusted.
Trace of the Covariance Matrix = 3
Condition Number of the Covariance Matrix = 5,806,065
Determinant of the Covariance Matrix = 0.0
Rank of the Covariance Matrix = 150
Trace of the Correlation Matrix = 150
Trace of the Partial Correlation Matrix = -150
PDB file with B-factors replaced by residue RMSDs: ss_50_RMSF_edited.pdb
The DVPs (PCs) from the 3 different models were calculated using:
Standard dot product (dp), normed dp, weighted dp (by eigenvalue), and weighted normed dp
Subspace analysis was done comparing the top vector spaces from the 3 different models.
Comparators include RMSIP and Principle Angles, for the essential subspace and iterated comparisons from dim
1 to 5
Additional log files can be found in the /SSA directory tree.


PERFORMING dpPCA, Computing Top 3 modes.

Residue Pair list:  residue_pairs.txt
Number of residues pairs: 5
No coordinate outliers were adjusted.
Trace of the Covariance Matrix = 0.000
Condition Number of the Covariance Matrix = 51
Determinant of the Covariance Matrix = 0
```

```
Rank of the Covariance Matrix = 5
Trace of the Correlation Matrix = 5
Trace of the Partial Correlation Matrix = -5

MEANs and STANDARD DEVIATIONs for the Residue Pair Distances:

Res1        Res2            Mean            Std_Dev
A225        A294            32.651          0.000
A294        A525            41.435          0.000
A325        A525            44.517          0.002
A525        A795            105.495         0.001
A525        B52             80.534          0.000

The DVPs (PCs) from the 3 different models were calculated using:
Standard dot product (dp), normed dp, weighted dp (by eigenvalue), and weighted normed dp.
Subspace analysis was done to compare the top vector spaces from the 3 different models.
Comparators include RMSIP and Principle Angles, for the essential subspace and iterated comparisons from dim
1 to 3
Additional log files can be found in the /SSA directory tree.

Performing Cartesian Mode Visualization on Top 3 cPCA modes.
Sets of 20 structures were generated to animate each selected cPCA mode, for the COV, CORR, and PCORR PCA
models.
Atoms of each residue were perturbed along the mode eigenvector using a sine function ranging from 0 to 2PI.
A PyMol(TM) script was generated for each mode to play the mode structures as a movie.
MODE AMPLITUDE = 2.500

Analysis completed: 2016-08-15 07:07:13
----------------------------------------------------------------------------------------------------
```

B. Sample PDB READ Log file:

```
1A6N.pdb
1A6N_froda_00000001.pdb
1A6N_froda_00000002.pdb
1A6N_froda_00000003.pdb
1A6N_froda_00000004.pdb
1A6N_froda_00000005.pdb
1A6N_froda_00000006.pdb
1A6N_froda_00000007.pdb
1A6N_froda_00000008.pdb
1A6N_froda_00000009.pdb
1A6N_froda_00000010.pdb
1A6N_froda_00000011.pdb
1A6N_froda_00000012.pdb
1A6N_froda_00000013.pdb
1A6N_froda_00000014.pdb
1A6N_froda_00000015.pdb
1A6N_froda_00000016.pdb
1A6N_froda_00000017.pdb
1A6N_froda_00000018.pdb
1A6N_froda_00000019.pdb
1A6N_froda_00000020.pdb
1A6N_froda_00000021.pdb
1A6N_froda_00000022.pdb
1A6N_froda_00000023.pdb
1A6N_froda_00000024.pdb
1A6N_froda_00000025.pdb
```

C. Sample SSA Log File:

```
--------------------------------------------------------------------------------
Top_COV_Eigenvectors:
Rows: 150
Cols: 5
Top_CORR_Eigenvectors:
Rows: 150
Cols: 5

Output Directory: C:\\Users\\Charles\\workspace\\JED_2.0\\JED_Test\\Multi\\JED_RESULTS_TEST/cPCA/SSA/CORR_vs_PCORR/
Projections file written to: Projections_dim_5.txt
Cumulative overlaps 1 --> 2 file written to: CO_1_2_dim_5.txt
Cumulative overlaps 2 --> 1 file written to: CO_2_1_dim_5.txt
Principle Angles file written to: PAs_dim_5.txt
Cosine Products file written to: Cosine_Products_dim_5.txt
Vectorial sums of angles file written to: Vector_Sums_of_Angles_dim_5.txt

The Inner Products of each vector in subspace 1 with each vector in subspace 2 are:

     -0.995      0.015     -0.004      0.003     -0.009
      0.016      0.993     -0.002      0.011     -0.028
      0.003      0.001     -0.985     -0.003     -0.014
     -0.004      0.008      0.004     -0.978      0.067
      0.025     -0.040      0.001     -0.059     -0.946


The cumulative overlaps CO_5 for each vector in subspace 1 with all the vectors in subspace 2 are:
Vector  1           0.996
Vector  2           0.994
Vector  3           0.985
Vector  4           0.980
Vector  5           0.949

The cumulative overlaps CO_5 for each vector in subspace 2 with all the vectors in subspace 1 are:
Vector  1           0.996
Vector  2           0.994
Vector  3           0.985
Vector  4           0.979
Vector  5           0.949

The RMSIP score is 0.981

The principle angles (in degrees) are:
PA    1           4
PA    2           6
PA    3           10
PA    4           12
PA    5           19

The cosine products (in degrees) are:
CP    1           4
CP    2           7
CP    3           12
CP    4           16
CP    5           25

The vectorial sums of angles (in degrees) are:
VS    1           4
VS    2           7
VS    3           12
VS    4           17
VS    5           25

Maximum possible angle between two subspaces of this dimension is 201 degrees

Analysis completed: 2016-08-15 07:07:09
--------------------------------------------------------------------------------
```

D. Sample FSSA Iterated Log File:

```
-------------------------------------------------------------------------------
Output Directory:
C:\\Users\\Charles\\workspace\\JED_2.0\\JED_Test\\Multi\\JED_RESULTS_TEST/cPCA/SSA/CORR_vs_PCORR/
Principle Angle Spectra file written to: Iterated_PAs.txt
RMSIPs file written to: Iterated_RMSIPs.txt


RMSIPs:
Dim   1           0.995
Dim   2           0.995
Dim   3           0.991
Dim   4           0.988
Dim   5           0.981

The PA spectra for the range of subspaces are:

    6    0    0    0    0
    5    7    0    0    0
    5    6   10    0    0
    5    6   10   12    0
    4    6   10   12   19


Analysis completed: 2016-08-15 07:07:09
-------------------------------------------------------------------------------
```
