# Supplementary material for: JED: a Java Essential Dynamics Program for comparative analysis of protein trajectories
Source: BMC Bioinformatics. 2017 May 25;18:271. doi: 10.1186/s12859-017-1676-y (PMC5445469; doi:10.1186/s12859-017-1676-y)
Supplement: Supplementary file 2 — Supporting Information. Figure S1. Example results from cPCA using the R matrix for native. Figure S2: Example results from cPCA using the R matrix for mutant. Figure S3. Subspace comparison between native and mutant cPCA results Figure S4: Example results from cPCA using the P matrix for native. Figure S5: Example results from cPCA using the P matrix for mutant. Figure S6: Selection of residue-pair distances. Figure S7. Subspace comparison between native and mutant dPCA results. Figure S8: Example results from dPCA using the Q matrix for native and mutant. Figure S9: Free energy surfaces based on all pairwise combinations of the top three PC-modes based on pooling the native and mutant trajectories. Table S1: Subspace comparison between all possible pairs of Q-, R- and P-matrices using cPCA for native and mutant. Table S2: A twenty dimensional subspace comparison between native and native for each of the Q-, R- and P-matrices using cPCA. Table S3: A twenty dimensional subspace comparison between all possible pairs of Q-, R- and P-matrices using dPCA for native and mutant. Table S4: A twenty dimensional subspace comparison between native and native for each of the Q-, R- and P-matrices using dPCA. Table S5: Same as Table S4 expect a 2 dimensional subspace is being compared. (PDF 4690 kb) [file 12859_2017_1676_MOESM2_ESM.pdf]

Supporting Information

JED: A Java Essential Dynamics Program for Comparative Analysis of Protein Trajectories

Charles C. David[1†]*, E. R. Azhagiya Singam[2] and Donald J. Jacobs[2]*

[1]Department of Bioinformatics and Genomics, [2]Department of Physics and Optical Science, University of North Carolina at Charlotte, USA

†Current Address: The New Zealand Institute for Plant & Food Research Limited, Lincoln, NZ

*Correspondence: charles.david@plantandfood.co.nz or djacobs1@uncc.edu

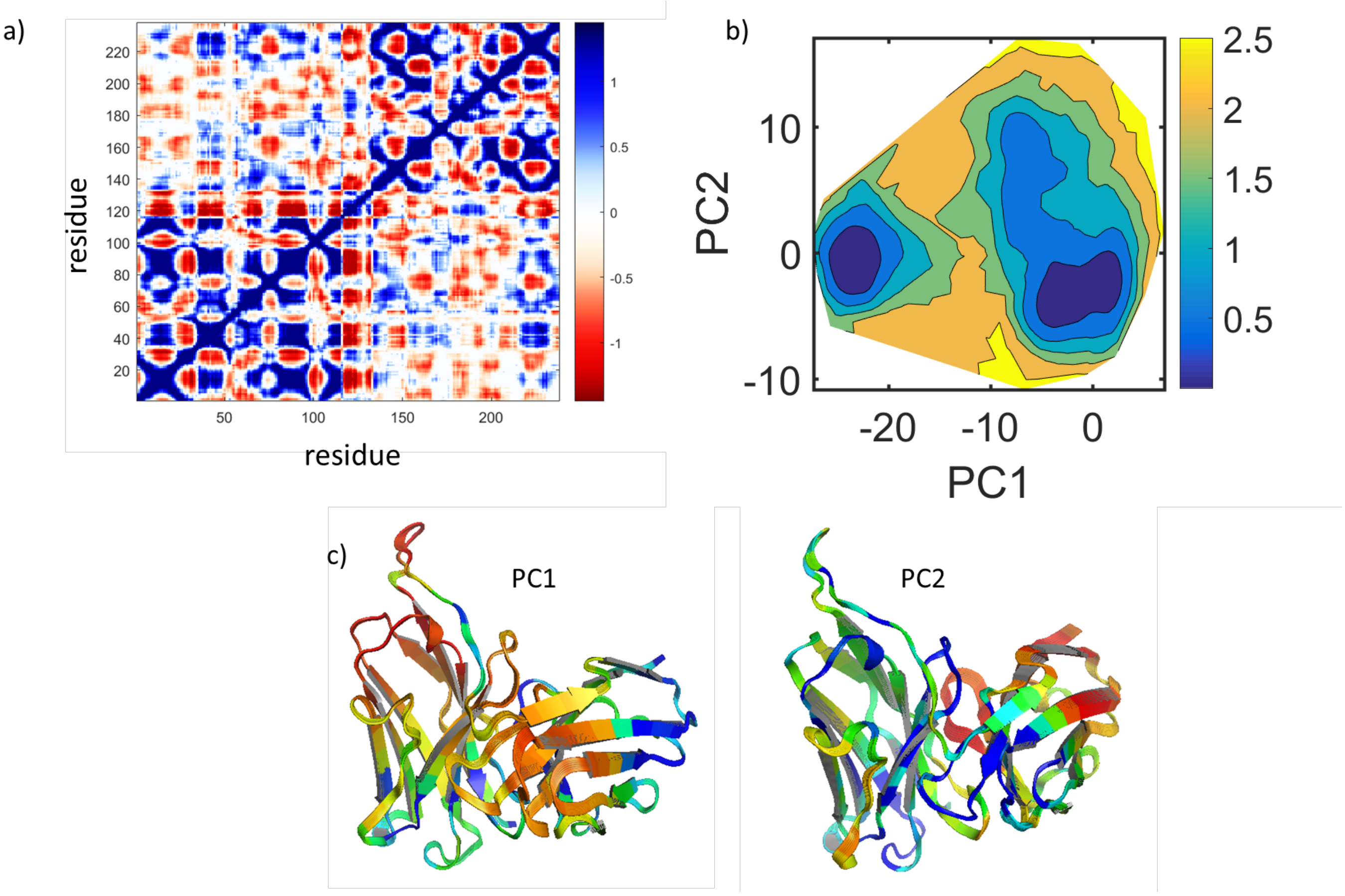

Figure S1: a) R matrix as obtained from cPCA of the native protein. b) Free energy surface as obtained from the first two principal components. c) The displacements along PC1 and PC2 are visualized and colored according to their RMSF of each residues using Pymol,

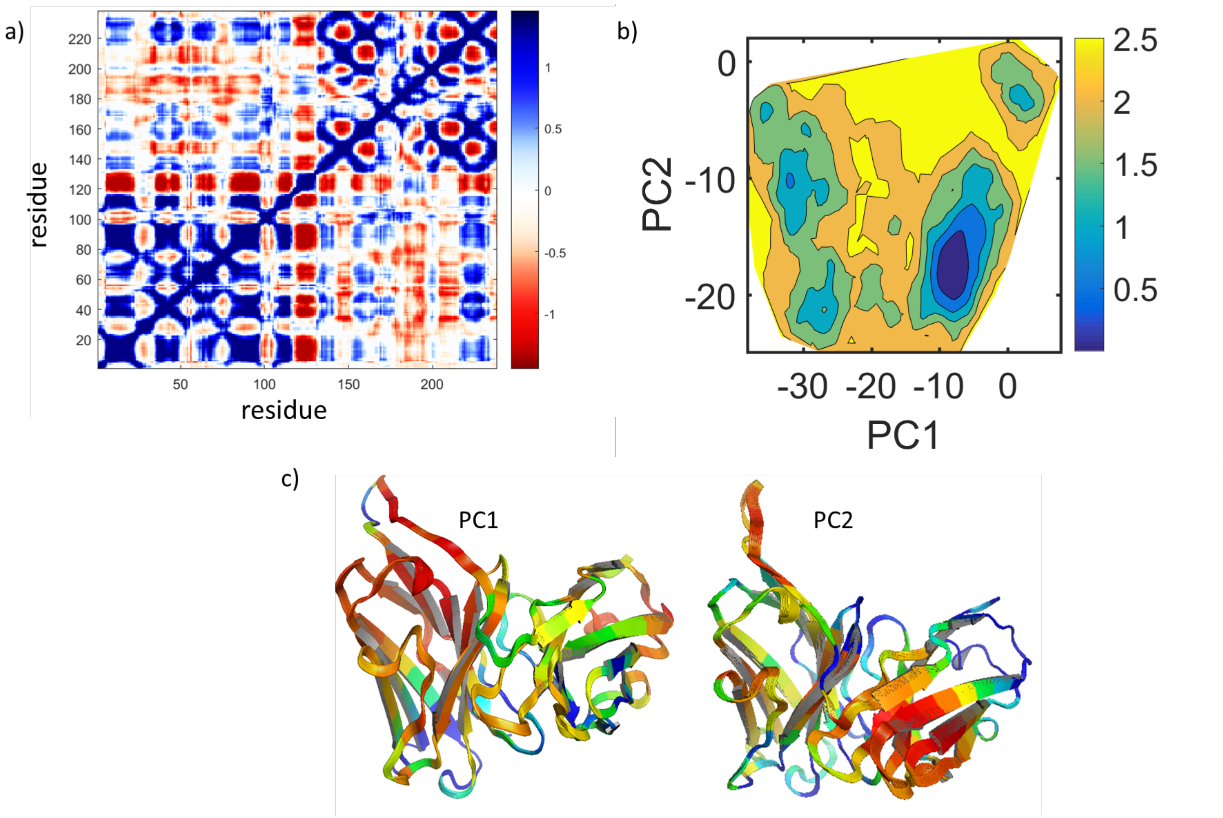

Figure S2: a) R matrix as obtained from cPCA of the mutant. b) Free energy surface as obtained from the first two principal components. c) The displacements along PC1 and PC2 are visualized and colored according to their RMSF of each residues using Pymol.

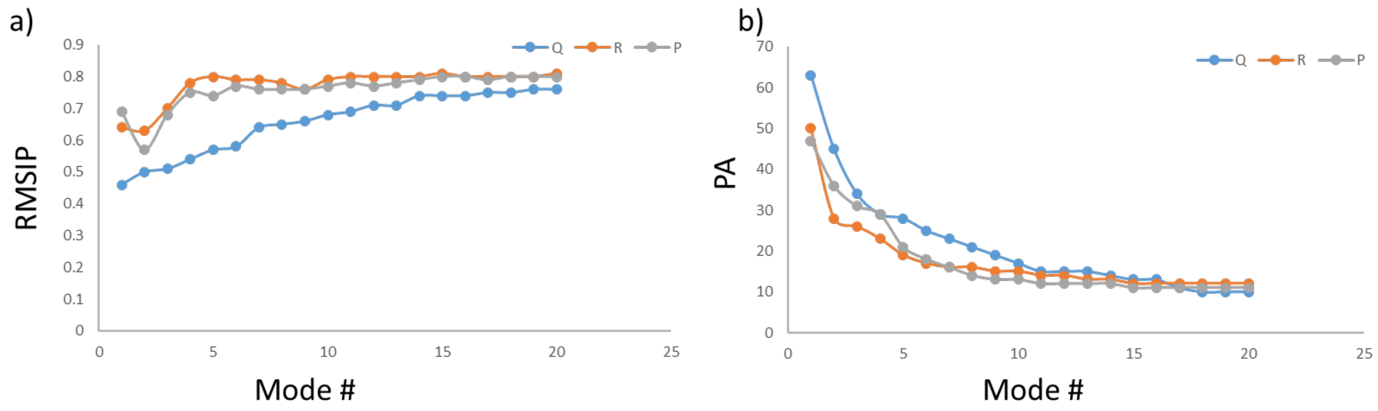

Figure S3. Subspace comparison between native and mutant proteins using a) RMSIP and b) principal angle as a function of subspace dimension as obtained from cPCA.

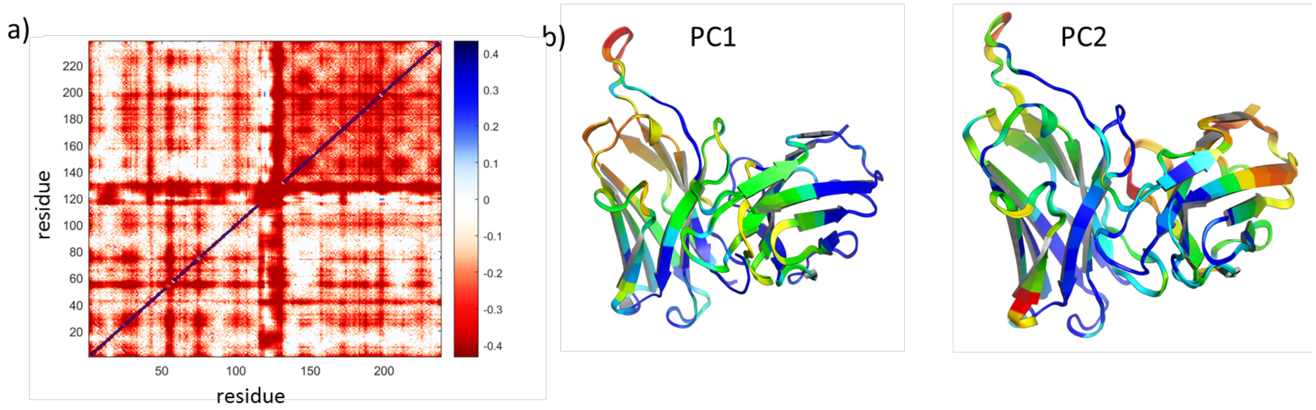

Figure S4: a) P matrix as obtained from the cPCA of the native protein. b) The displacements along PC1 and PC2 are visualized and colored according to RMSF using Pymol,

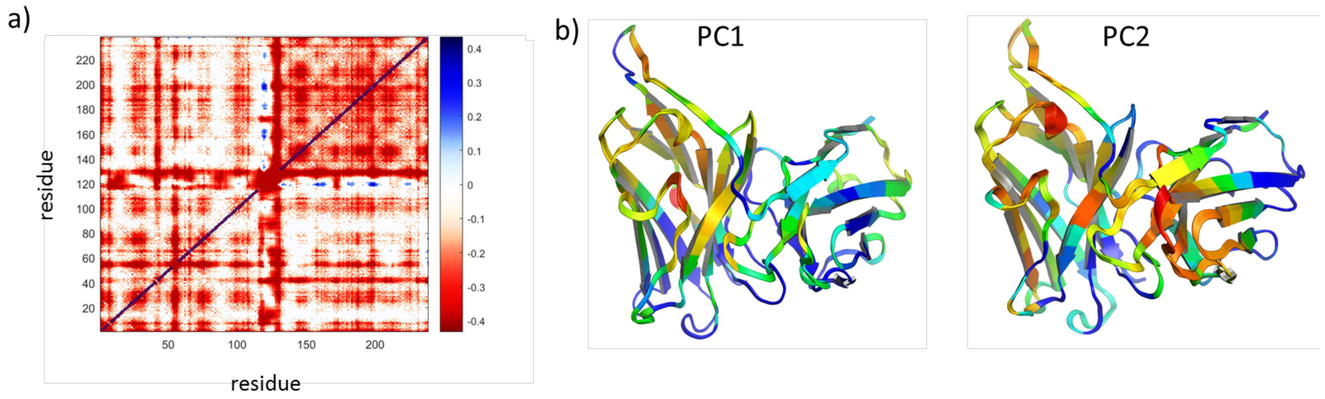

Figure S5: a) P matrix as obtained from cPCA of the mutant. b) The displacements along PC1 and PC2 are visualized and colored according to RMSF using Pymol.

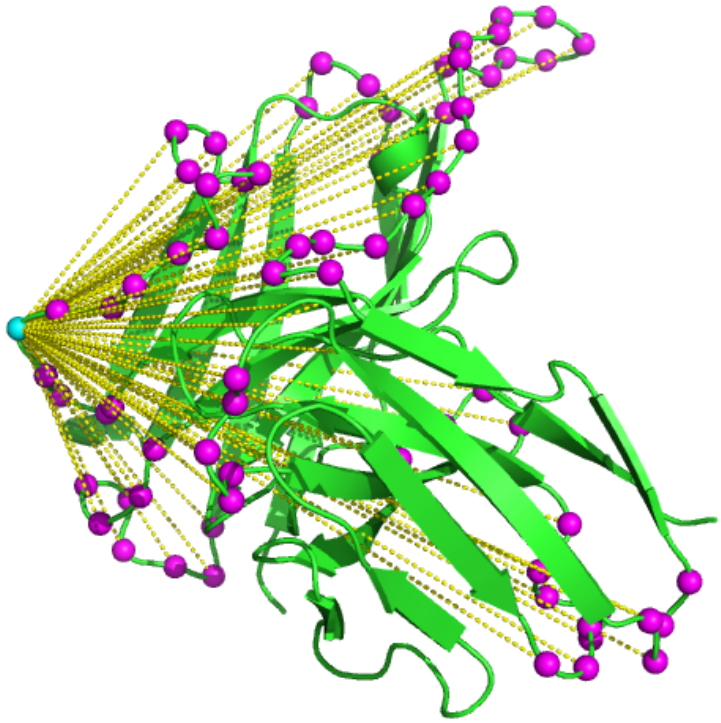

Figure S6: Alpha carbons (beads) defines the residue-pair distances from the different loop regions (marked in magenta) to residue 56 (cyan)

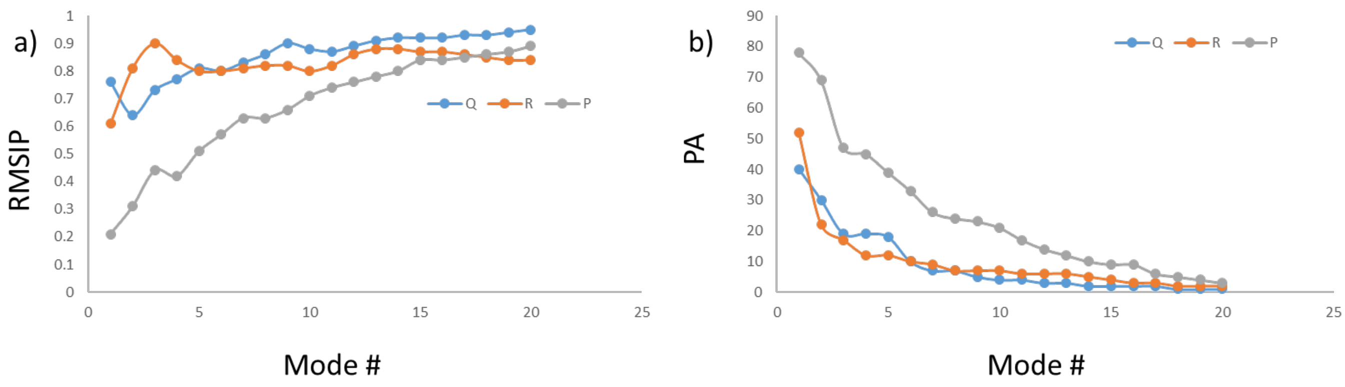

Figure S7. Subspace comparison between native and mutant proteins using a) RMSIP and b) principal angle as a function of subspace dimension as obtained from dpPCA.

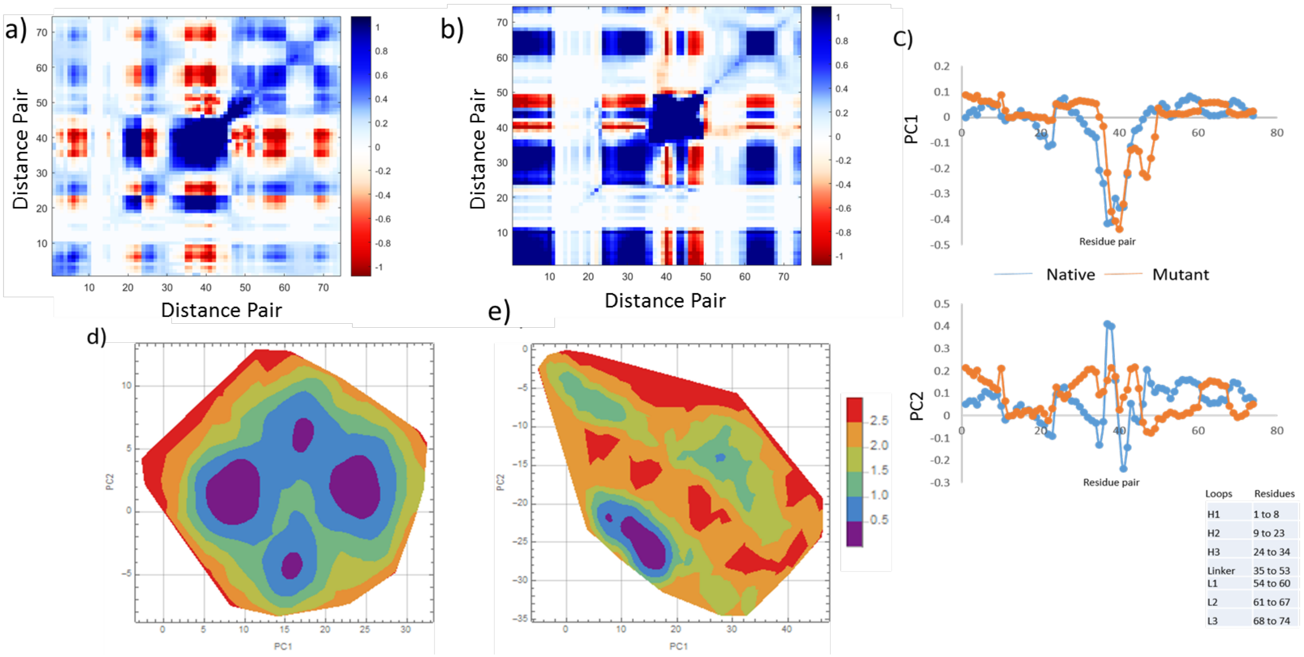

Figure S8: a) and b) Covariance Matrix (Q) as obtained from the dpPCA of native and mutant respectively. c) Eigenvector component of first two principal components of native and mutant. d) and e) Free energy surface as obtained from the first two principal components of native and mutant respectively.

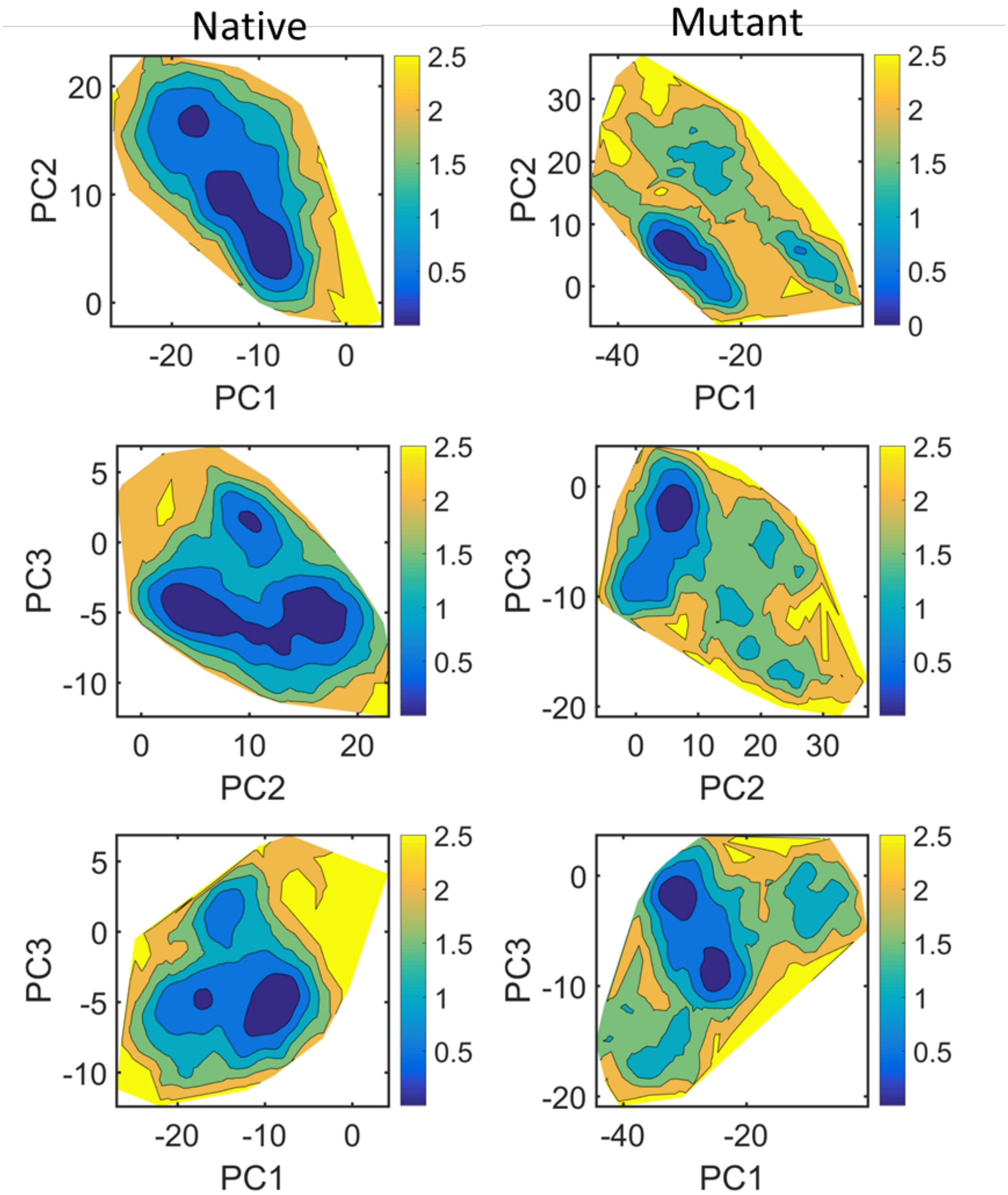

Figure S9: Free energy surface obtained from all pairwise combinations of the top three PC-modes based on pooling the native and mutant trajectories.

Table S1: Out of a 714 dimensional space from cPCA, a summary of how three different 20 dimensional subspaces based on the eigenvectors from the Q-, R- and P-matrices overlap and how they align is given. The principal angles are ordered from smallest to largest, listing the smallest angle necessary to rotate about the n-th axis to maximally align the two subspaces. Each next axis of rotation requires the same amount of rotation or more. A principal angle of 30 degrees or less represents very good similarity, and 60 degrees or larger represents high dissimilarity. Angles between 30 to 60 degrees do not represent either very different or similar characteristics, with 45 degrees representing 50% similarity/dissimilarity. RMSIP is a single number that represents overall overlap between the two 20-dimensional subspaces being compared.

D=20	Native			Mutant		
n-th axis	R-P	Q-R	Q-P	R-P	Q-R	Q-P
1	11	18	17	10	20	18
2	12	19	18	11	21	20
3	13	24	24	12	26	26
4	14	31	26	12	38	31
5	15	32	29	13	42	41
6	17	34	33	13	43	45
7	17	38	37	15	51	50
8	18	43	38	16	52	51
9	19	49	40	16	54	54
10	20	53	45	18	61	61
11	21	56	52	18	66	66
12	23	60	62	21	69	70
13	24	67	66	21	70	71
14	26	77	71	23	79	77
15	28	80	76	25	83	79
16	33	82	80	27	84	82
17	34	83	82	37	84	83
18	39	85	85	40	87	85
19	46	86	86	43	88	88
20	56	90	88	71	89	90
RMSIP	0.9	0.616	0.643	0.906	0.552	0.561

Table S2: Out of a 714 dimensional space from cPCA, a summary of how three different 20 dimensional subspaces based on the eigenvectors from the Q-, R- and P-matrices overlap and how they align is given. In this case the comparison is between the native and mutant proteins with respect to the same type of quantity. Again, the principal angles are ordered from smallest to largest, listing the smallest angle necessary to rotate about the n-th axis to maximally align the two subspaces. Each next axis of rotation requires the same amount of rotation or more. A principal angle of 30 degrees or less represents very good similarity, and 60 degrees or larger represents high dissimilarity. Angles between 30 to 60 degrees do not represent either very different or similar characteristics, with 45 degrees representing 50% similarity/dissimilarity. RMSIP is a single number that represents overall overlap between the two 20-dimensional subspaces being compared.

n-th axis	Q	R	P
1	9.85	11.93	10.84
2	11.99	12.68	11.42
3	14.69	13.48	11.82
4	15.85	15.52	14.53
5	16.62	17.01	17.01
6	17.91	18.53	18.12
7	18.59	19.61	21.23
8	23.52	21.71	22.39
9	28.42	23.6	26.31
10	30.19	26.74	28.15
11	32.97	30.33	31.78
12	38.03	33.87	36.15
13	43.53	34.23	39.06
14	54.49	41.72	40.84
15	64.78	44.88	47.54
16	66.15	48.92	53.07
17	74.26	59.47	57.93
18	76.57	67.38	70.37
19	80.96	76.55	78.56
20	83.5	81.93	88.51
RMSIP	0.76	0.81	0.80

Table S3: Out of a 74 dimensional space from dpPCA, a summary of how three different 20 dimensional subspaces based on the eigenvectors from the Q-, R- and P-matrices overlap and how they align is given. The principal angles are ordered from smallest to largest, listing the smallest angle necessary to rotate about the n-th axis to maximally align the two subspaces. Each next axis of rotation requires the same amount of rotation or more. A principal angle of 30 degrees or less represents very good similarity, and 60 degrees or larger represents high dissimilarity. Angles between 30 to 60 degrees do not represent either very different or similar characteristics, with 45 degrees representing 50% similarity/dissimilarity. RMSIP is a single number that represents overall overlap between the two 20-dimensional subspaces being compared.

D=20		Native			Mutant	
n-th axis	R-P	Q-R	Q-P	R-P	Q-R	Q-P
1	11	18	17	10	20	18
2	12	19	18	11	21	20
3	13	24	24	12	26	26
4	14	31	26	12	38	31
5	15	32	29	13	42	41
6	17	34	33	13	43	45
7	17	38	37	15	51	50
8	18	43	38	16	52	51
9	19	49	40	16	54	54
10	20	53	45	18	61	61
11	21	56	52	18	66	66
12	23	60	62	21	69	70
13	24	67	66	21	70	71
14	26	77	71	23	79	77
15	28	80	76	25	83	79
16	33	82	80	27	84	82
17	34	83	82	37	84	83
18	39	85	85	40	87	85
19	46	86	86	43	88	88
20	56	90	88	71	89	90
RMSIP	0.9	0.616	0.643	0.906	0.552	0.561

Table S4: Out of a 74 dimensional space from dpPCA, a summary of how three different 20 dimensional subspaces based on the eigenvectors from the Q-, R- and P-matrices overlap and how they align is given. In this case the comparison is between the native and mutant proteins with respect to the same type of quantity. Again, the principal angles are ordered from smallest to largest, listing the smallest angle necessary to rotate about the n-th axis to maximally align the two subspaces. Each next axis of rotation requires the same amount of rotation or more. A principal angle of 30 degrees or less represents very good similarity, and 60 degrees or larger represents high dissimilarity. Angles between 30 to 60 degrees do not represent either very different or similar characteristics, with 45 degrees representing 50% similarity/dissimilarity. RMSIP is a single number that represents overall overlap between the two 20-dimensional subspaces being compared.

n-th axis	Q	R	P
1	1.06	2.13	2.74
2	1.43	3.56	3.53
3	2.53	3.9	5.07
4	2.67	4.9	5.7
5	3.09	6.6	7.54
6	3.25	7.95	8.34
7	3.57	8.69	9.6
8	3.85	11.16	11.02
9	4.45	12.99	12.3
10	5.21	15.96	12.71
11	6.21	20.09	13.67
12	7.33	22.25	16.91
13	9.38	27.05	21.41
14	10.16	34.83	22.85
15	11.04	39.79	27.82
16	14.57	59.46	33.64
17	17.28	71.12	42.11
18	24.91	76.53	55
19	51.07	86.76	64.78
20	58.16	89.27	83.15
RMSIP	0.95	0.84	0.89

Table S5: Same as S4 expect the size of the vector space being compared is only 2 dimensional.

n-th axis	Q	R	P
1	30.15	22.43	15.11
2	73.52	47.24	45.37
RMSIP	0.64	0.81	0.84
