# Supplementary material for: JED: a Java Essential Dynamics Program for comparative analysis of protein trajectories
Source: BMC Bioinformatics. 2017 May 25;18:271. doi: 10.1186/s12859-017-1676-y (PMC5445469; doi:10.1186/s12859-017-1676-y)
Supplement: Supplementary file 3 — Movies showing mode 1 and mode 2 of all the modes obtained from the JED program. (PPT 58883 kb) [file 12859_2017_1676_MOESM3_ESM.ppt]

## Slide 1
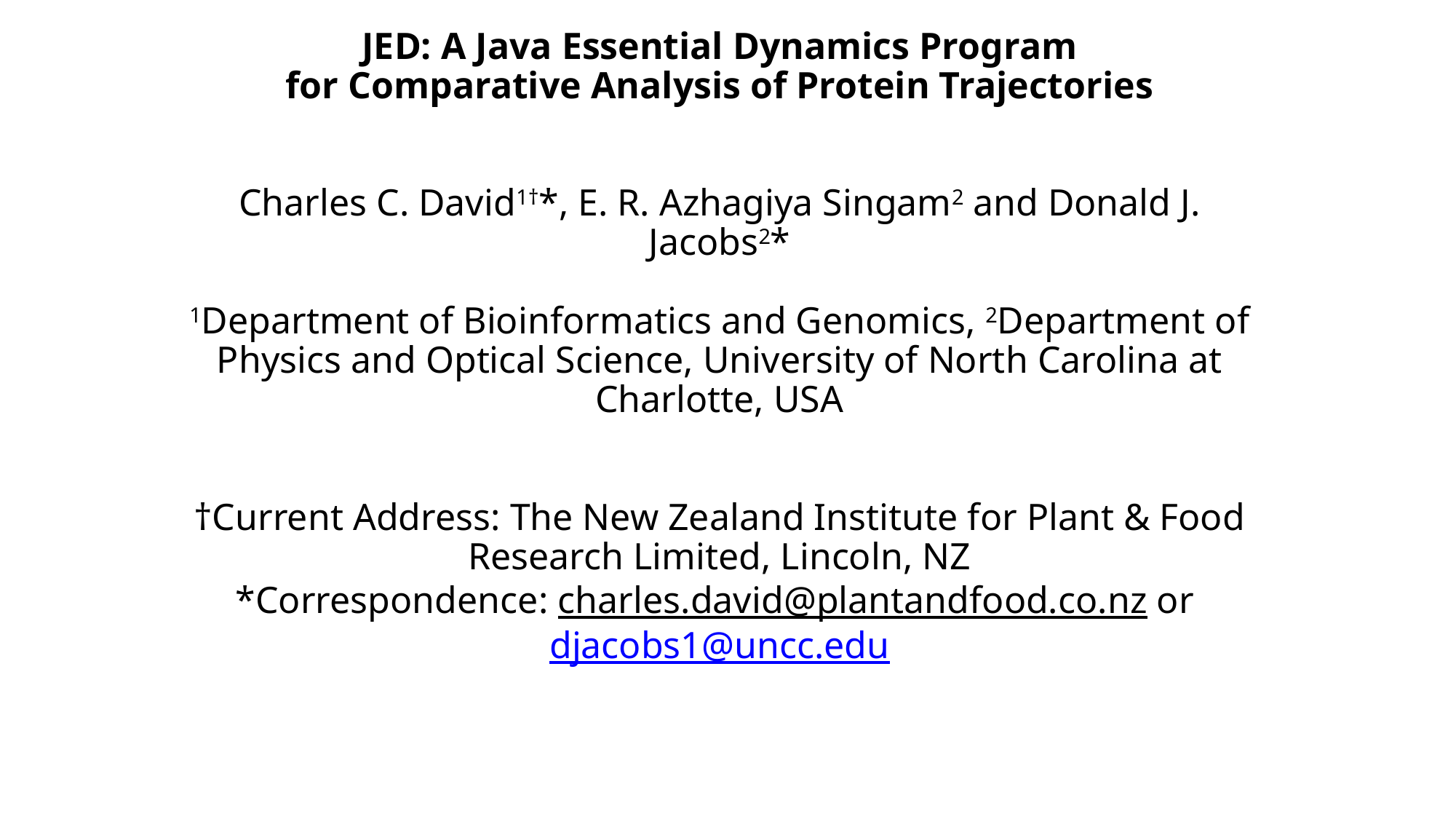

# JED: A Java Essential Dynamics Program for Comparative Analysis of Protein Trajectories Charles C. David1†*, E. R. Azhagiya Singam2 and Donald J. Jacobs2*   1Department of Bioinformatics and Genomics, 2Department of Physics and Optical Science, University of North Carolina at Charlotte, USA   †Current Address: The New Zealand Institute for Plant & Food Research Limited, Lincoln, NZ *Correspondence: charles.david@plantandfood.co.nz or djacobs1@uncc.edu

## Slide 2
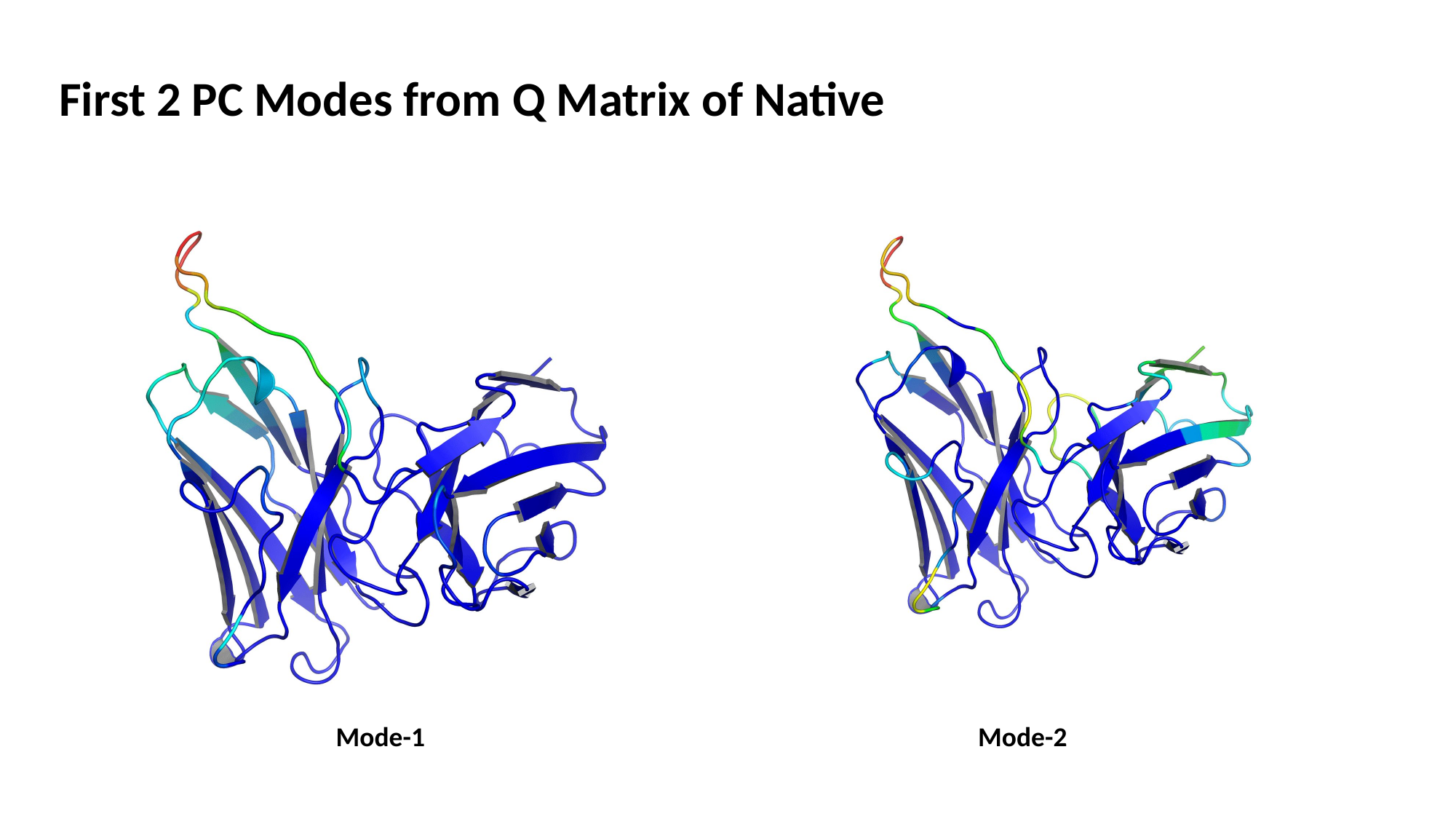

First 2 PC Modes from Q Matrix of Native
Mode-1
Mode-2

## Slide 3
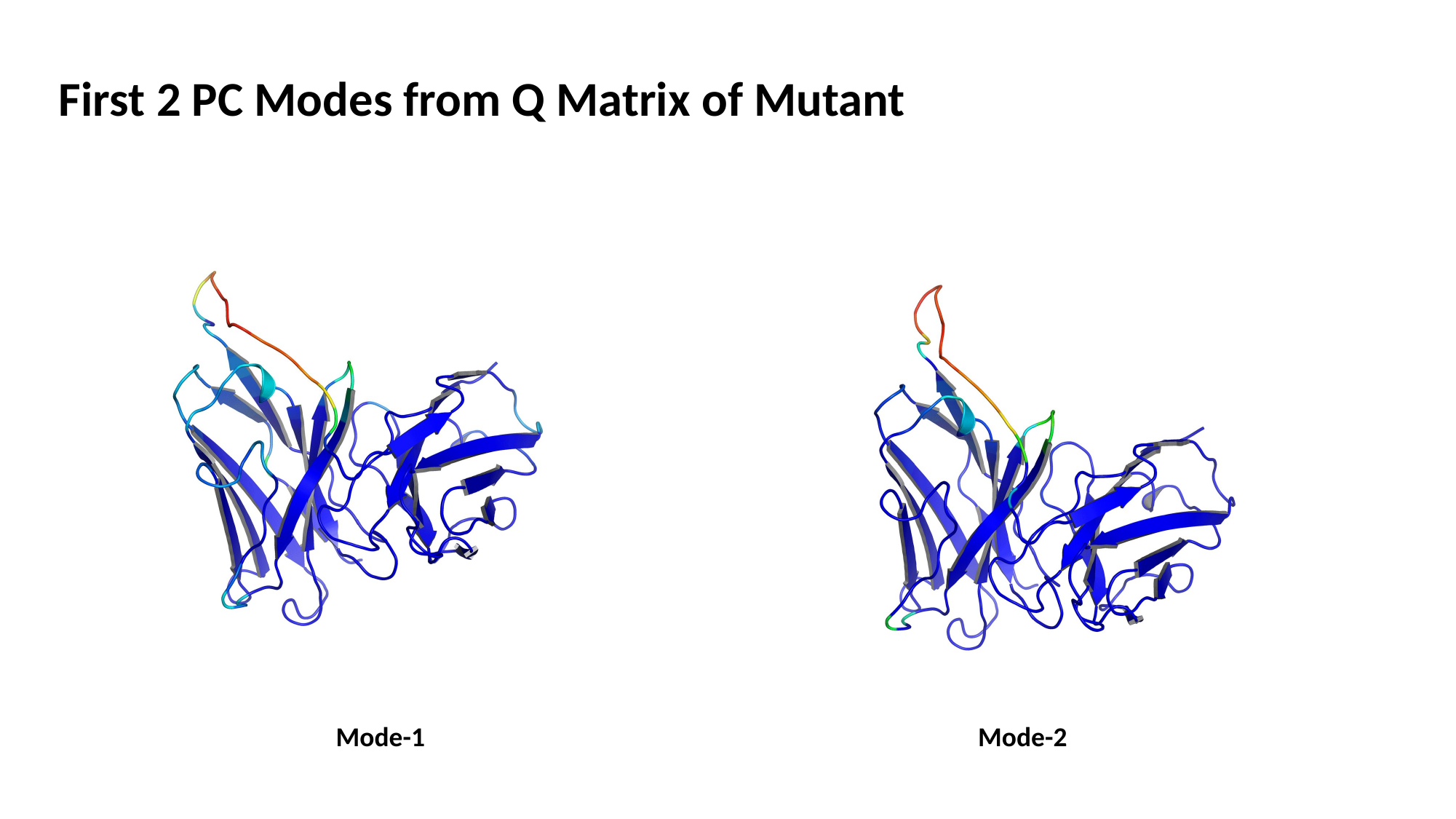

First 2 PC Modes from Q Matrix of Mutant
Mode-1
Mode-2

## Slide 4
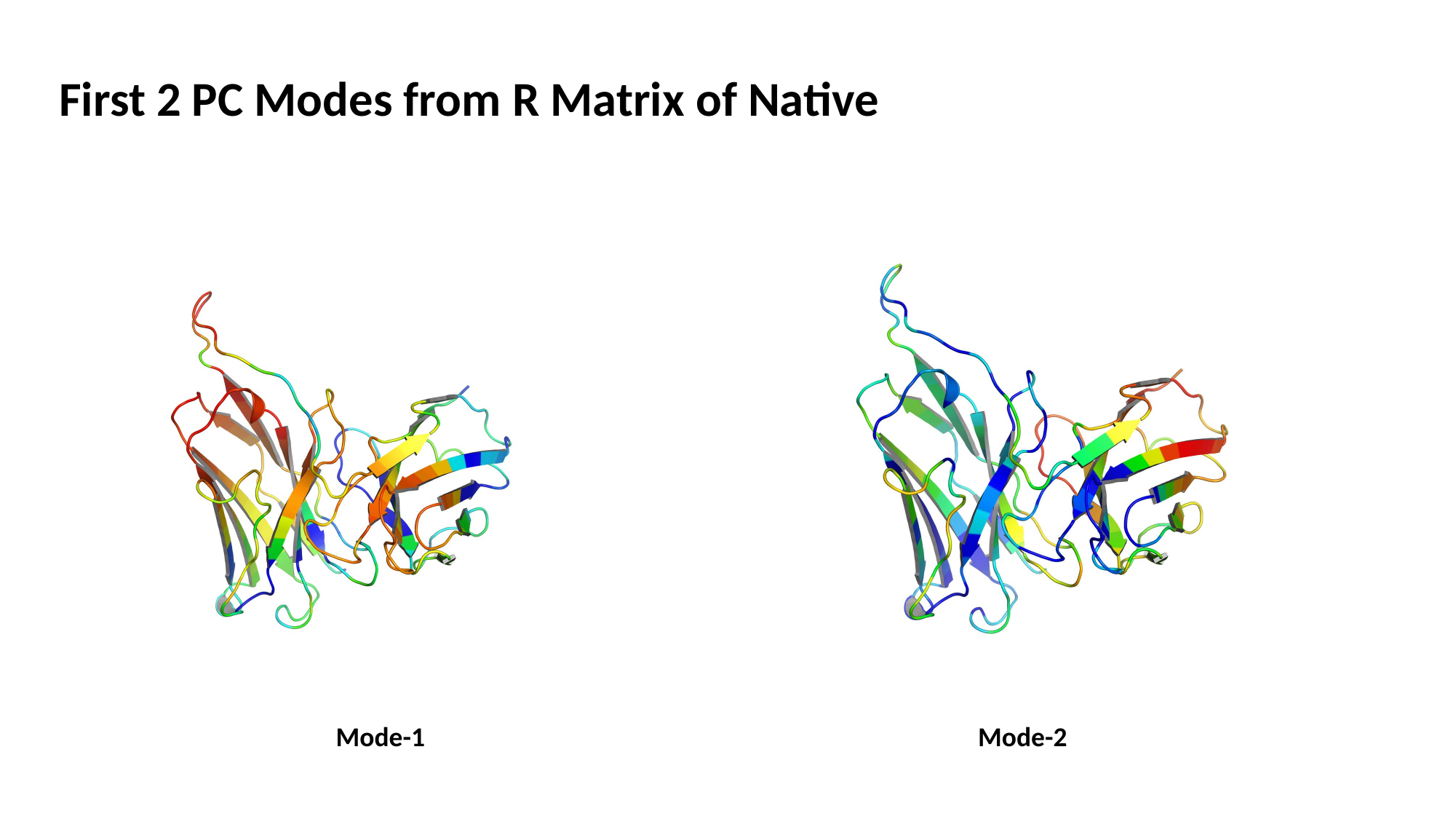

First 2 PC Modes from R Matrix of Native
Mode-1
Mode-2

## Slide 5
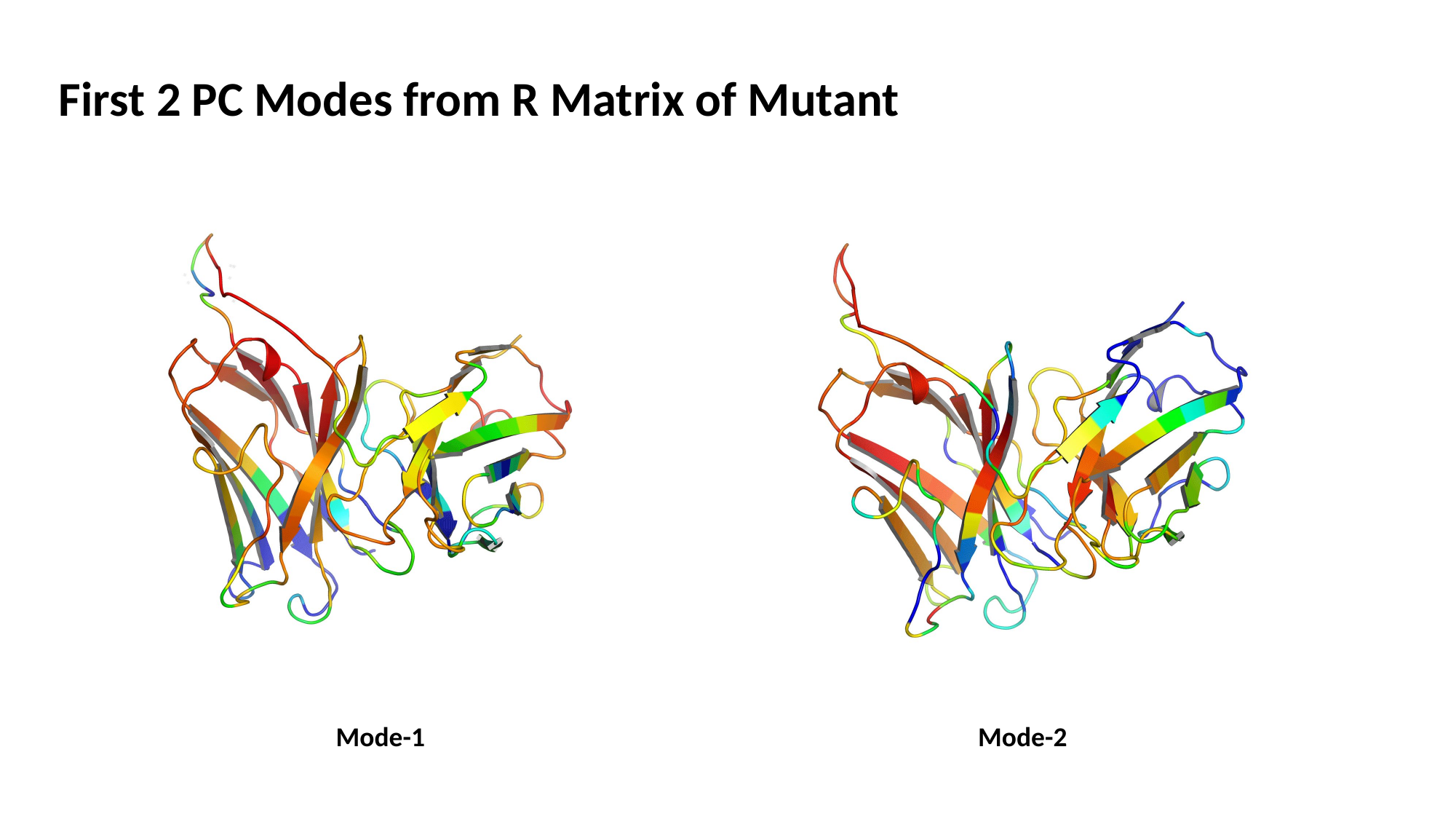

First 2 PC Modes from R Matrix of Mutant
Mode-1
Mode-2

## Slide 6
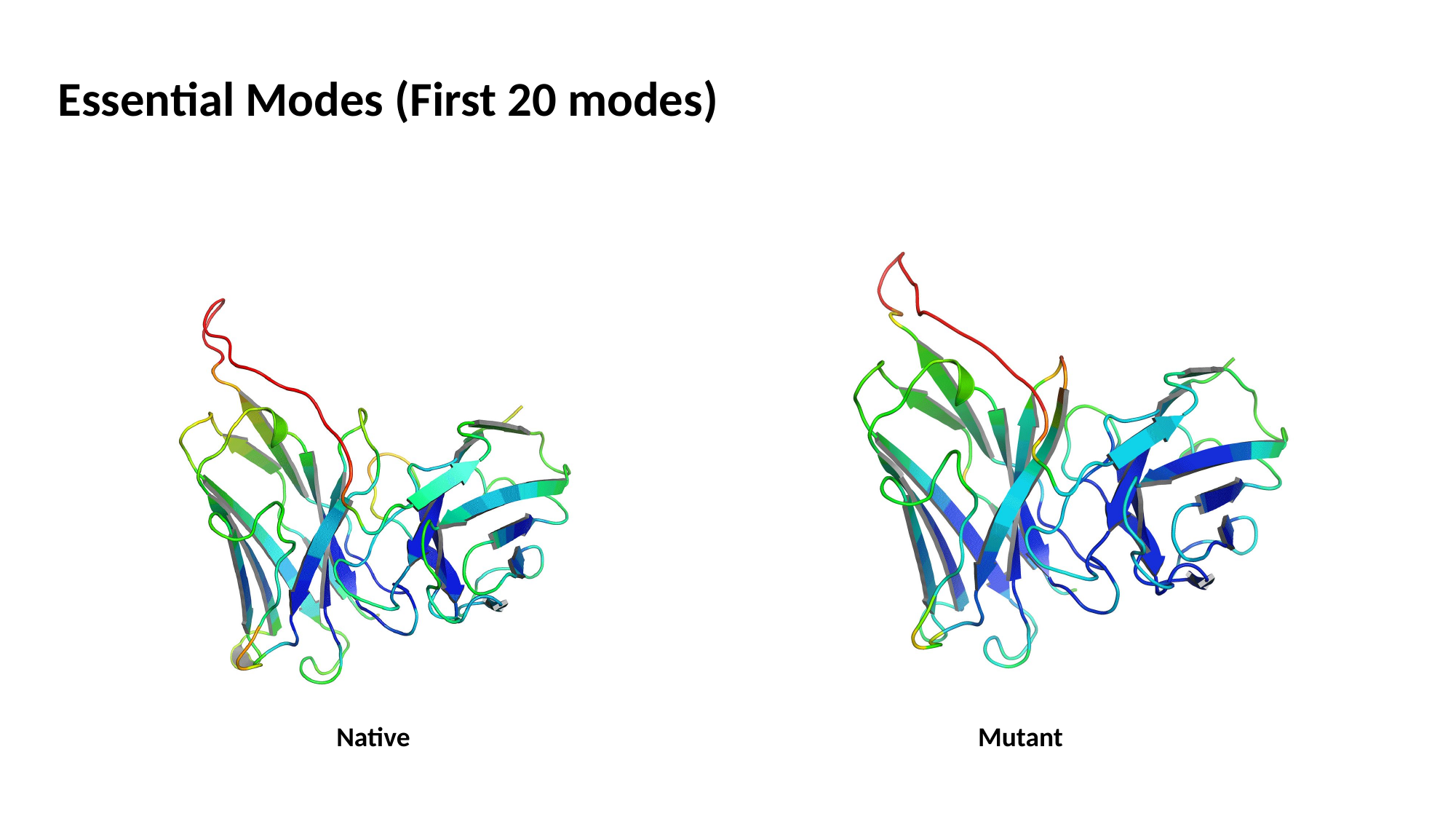

Essential Modes (First 20 modes)
Native
Mutant
